# Drought-tolerant and drought-sensitive genotypes of maize (*Zea mays* L.) differ in contents of endogenous brassinosteroids and their drought-induced changes

**DOI:** 10.1371/journal.pone.0197870

**Published:** 2018-05-24

**Authors:** Lenka Tůmová, Danuše Tarkowská, Kateřina Řehořová, Hana Marková, Marie Kočová, Olga Rothová, Petr Čečetka, Dana Holá

**Affiliations:** 1 Department of Genetics and Microbiology, Faculty of Science, Charles University, Prague, Czech Republic; 2 Laboratory of Growth Regulators, Centre of the Region Haná for Biotechnological and Agricultural Research, Institute of Experimental Botany, Academy of Sciences of the Czech Republic, v.v.i. and Palacký University, Olomouc, Czech Republic; Estacion Experimental del Zaidin, SPAIN

## Abstract

The contents of endogenous brassinosteroids (BRs) together with various aspects of plant morphology, water management, photosynthesis and protection against cell damage were assessed in two maize genotypes that differed in their drought sensitivity. The presence of 28-norbrassinolide in rather high quantities (1–2 pg mg^-1^ fresh mass) in the leaves of monocot plants is reported for the first time. The intraspecific variability in the presence/content of the individual BRs in drought-stressed plants is also described for the first time. The drought-resistant genotype was characterised by a significantly higher content of total endogenous BRs (particularly typhasterol and 28-norbrassinolide) compared with the drought-sensitive genotype. On the other hand, the drought-sensitive genotype showed higher levels of 28-norcastasterone. Both genotypes also differed in the drought-induced reduction/elevation of the levels of 28-norbrassinolide, 28-norcastasterone, 28-homocastasterone and 28-homodolichosterone. The differences observed between both genotypes in the endogenous BR content are probably correlated with their different degrees of drought sensitivity, which was demonstrated at various levels of plant morphology, physiology and biochemistry.

## Introduction

The economic losses in crop production due to drought are quite substantial and will undoubtedly further increase with the expected climate changes. To prepare for these changes, various new agricultural technologies are tried and utilised [1, 2]. Some attention has been paid to the application of diverse chemical compounds such as polyethylene glycol (PEG), amino acids, antioxidants, phytohormones, minerals, volatile organic compounds, *etc*. [3]. A group of steroidal phytohormones called brassinosteroids (BRs) has been included among these compounds. Indeed, BRs seem to be destined for agricultural practice due to the fact they are non-toxic, non-mutagenic and environmentally friendly, as well as their effectivity at low concentrations, ease of application and the possibility of artificially synthesising them on a commercial scale [4–6].

Studies on the impact of BRs on drought-stressed plants began almost 25 years ago [7], and since that time, more than 90 papers on this topic have appeared in various scientific journals or books. BRs are generally regarded as positive regulators of the plant drought response, and the elevation of their contents in plants by exogenous application is often accompanied by an improvement in drought resistance. The studies presenting some data on the response of BR-treated plants or mutants in BR biosynthesis/perception to drought greatly vary regarding the overall design of experiments, plant cultivation conditions, examined species, developmental stage of plants, *etc*. However, these studies usually have one attribute in common: their authors analysed only one genotype/cultivar of the respective plant species. Although some papers deal with more than one genotype, the drought sensitivity/resistance of these genotypes is often unspecified. This fact significantly limits our understanding of the relationship between BRs and drought resistance and could limit the potential practical application of these compounds.

The data on the role of BRs in plant drought response that are currently available from the few studies that have been performed with genotypes of known drought sensitivity are not very conclusive. Logically, any comparison of the impact of exogenously applied BRs on drought-resistant/sensitive genotypes should reveal the BR-induced changes particularly in the sensitive genotypes, because the resistant genotypes should experience less intensive drought effects. This should be similar to the situation observed for the BRs exogenously applied to plants exposed to severe or mild/moderate drought, BRs always have a greater effect on more strongly stressed plants [8–11]. Such response of drought-sensitive and drought-resistant genotypes to BRs has indeed been observed by [12] and [13] in sorghum exposed to PEG. However, a more-or-less similar response of drought-sensitive and drought-resistant genotypes was demonstrated in wheat stressed by the cessation of watering or subjected to PEG or mannitol treatment [14–16], as well as in tomato [17]. There are also some cases where the drought-tolerant genotype showed a more pronounced response to BRs than the drought-sensitive one, this been reported for PEG-stressed maize [18–20] or rice [21]. Thus, the situation is not so simple and probably depends on plant species as well as on a mechanism that is responsible for drought resistance/sensitivity of the respective genotype. In addition, most of the authors of the abovementioned studies simulated drought by the application of PEG. This certainly causes *osmotic* stress, but the application of PEG induces a specific stress level very rapidly and very strongly, thus evading the natural course of drought response with its gradual changes. Therefore, the results obtained from such studies might not mimic drought situations occurring in nature.

Moreover, although diverse aspects of plant biology have been examined during research on the role of BRs in plant drought response, most scientists have focused only on the possible alleviation of stress symptoms by exogenously applied BRs. Almost no one has pursued the possibility that drought *per se* could induce changes in the content of *endogenous* BRs. Indeed, such analysis has rarely been performed for any type of stress and only three studies give some information on this topic. Jäger *et al*. [22] assessed the content of one bioactive BR (castasterone, CS) in the drought-stressed pea. They found that the exposure of their experimental plants to adverse environment increased the content of this BR, but only non-significantly. Gruszka *et al*. [23], who examined barley plants subjected to water deficiency, reported that drought induced the levels of endogenous CS and 24-*epi*brassinolide (*epi*BL) but did not change the amounts of brassinolide (BL), 24-*epi*castasterone (*epi*CS) or 28-homocastasterone (homoCS). Finally, Pociecha *et al*. [24] assessed the CS content in two cultivars of cold-acclimated winter rye. The winter-resistant cultivar showed the same elevation of CS (approximately 2-fold) after 3 or 6 weeks of cultivation in cold, while the less winter-resistant cultivar was characterised by a 2-fold increase after 3 weeks and an additional increase after 6 weeks of cold acclimation.

We thus decided to compare the contents of various endogenous BRs in drought-resistant and drought-sensitive maize genotypes to examine their changes under drought stress. To our knowledge, such analysis has not been previously performed. Additionally, to determine the possible relationship between these changes and various processes that occur during plant drought response, we also examined several aspects of plant morphology and physiology. Because BRs are generally considered to play a positive role in plant stress resistance, we hypothesised that the resistant genotype could be characterised by higher levels of endogenous BRs than the sensitive genotype already under non-stress conditions. However, because the resistant genotype would not experience stress to such a degree as the other genotype, we also expected that the resistant genotype would not need to further elevate the BR contents when subjected to drought.

## Material and methods

### Plant material, experimental design and BR treatment

Two maize (*Zea mays* L.) inbred lines, drought-sensitive 2023 and drought-resistant CE704, were used for our experiments. The degree and mechanisms of their drought-sensitivity are described in [25, 26]. Kernels of both genotypes were obtained from the *CEZEA Maize Breeding Station* (Czech Republic) and were sown in pots (diameter 12 cm, height 12 cm, one plant per pot) filled with a mixture (15:1, v:v) of Garden Compost (*Agro CS*, Czech Republic) and Hawita Baltisches Uni 20 Tonsubstrat 1 (*Hawita*, Germany). The pots were placed in a naturally lit greenhouse located on the Faculty of Science campus at Charles University, Prague, Czech Republic (54°04'N, 14°25'E) under semi-controlled conditions (mean air temperature 24/16°C, mean relative air humidity 71/86% day/night, natural irradiance). All plants were sufficiently watered until 30 days after the date of sowing. At this time, all the plants had three fully developed leaves and the fourth leaf was visible from at least one half of its final length. They were then divided into four groups (experimental variants) according to genotype (2023 or CE704) and watering treatment (control, *i*.*e*., the 20% soil water content, or drought stress, *i*.*e*., the cessation of watering for 14 days resulting in the 3% soil water content at the end of the drought period). Each group consisted of 60 plants organised in a randomised plot design. This enabled us to use different plants for the determination of various parameters (*i*.*e*., we had separate groups of plants allocated for the assessments of i) the contents of BRs, ii) plant morphology, iii) gas exchange and the osmotic potential, iv) chlorophyll fluorescence and the contents of chlorophylls and carotenoids, and v) index of cell membrane injury (CMI), the contents of malondialdehyde (MDA), H_2_O_2_, proline, and the activities of ascorbate peroxidase (APX) and catalase (CAT). The fourth leaf (counting from the base) was utilised for all the measurements and samplings. The evaluation of plant morphology, gas exchange analysis, chlorophyll fluorescence measurements and determination of the photosynthetic pigment content and CMI were conducted immediately after the end of the experiments. For other parameters, the respective leaves were sampled at that time and kept at -80°C (in case of the leaf osmotic potential in -18°C) until their analyses. The exact number of biological replicates (3 to 12) for the individual parameters is always indicated in the legends to the respective figures/tables.

### Content of brassinosteroids

Samples were analysed for the BR contents according to the method described in [27]. Briefly, 50 mg of fresh maize tissue samples were homogenised to a fine consistency using 3-mm zirconium oxide beads and an MM 301 vibration mill at a frequency of 30 Hz for 3 min (*Retsch GmbH & Co*. *KG*, Haan, Germany). The samples were then extracted overnight with stirring at 4°C using a benchtop laboratory rotator Stuart SB3 (Bibby Scientific Ltd., Staffordshire, UK) after adding 1 mL ice-cold 60% acetonitrile and 10 pmol of [^2^H_3_]BL, [^2^H_3_]CS, [^2^H_3_]24-*epi*BL, [^2^H_3_]24-*epi*CS, [^2^H_3_]28-norBL, [^2^H_3_]28-norCS and [^2^H_3_]typhasterol as internal standards (OlChemIm Ltd., Olomouc, Czechia). The samples were further centrifuged, purified on polyamide SPE columns (Supelco, Bellefonte, PA, USA) and then analysed by UHPLC-MS/MS (Micromass, Manchester, UK). The data were analysed using Masslynx 4.1 software (Waters, Milford, MA, USA) and the BR contents were finally quantified by the standard isotope-dilution method [28]. Our study focused on the content of fifteen BRs: teasterone (TE), 28-norteasterone (norTE), typhasterol (TY), CS, *epi*CS, homoCS, 28-norcastasterone (norCS), BL, *epi*BL, 28-homobrassinolide (homoBL), 28-norbrassinolide (norBL), dolicholide (DL), 28-homodolicholide (homoDL), dolichosterone (DS) and 28-homodolichosterone (homoDS). Each experimental variant was represented by three biological replicates, where the level of each of these replicates was calculated as an arithmetic mean of two independent technical replicates. One biological replicate represents an individual leaf sampled at the end of the drought period and kept at -80°C until UHPLC-MS/MS analysis.

### The assessment of plant morphology and the measurements of the leaf osmotic potential

The height of plants was measured from the base of the shoot to the tip of the youngest leaf visible at the top whorl of leaves. The number of visible leaves was also counted. The area of individual leaves was calculated from their lengths and widths using a previously determined coefficients [26]. The total area of the photosynthetically active leaves was calculated as the sum of the area of all the leaves that were at least 50% green. Dry masses of individual leaves and the rest of shoot (their sum constitutes the total dry mass of shoot) and the total dry mass of roots were assessed after drying the respective parts of the plants for seven days at 80°C and weighing on analytical balances. The specific mass of the 4^th^ leaf was determined from ten leaf discs (diameter 6 mm) cut from the middle portion of the leaf blade, oven-dried at 80°C for two days and weighed on analytical balances with 0.1 mg precision.

For the measurements of the leaf osmotic potential, the samples of plant leaves were kept gently compressed in syringes sealed with Parafilm M till the time of measurements when the samples were thawed at 2°C. Approximately 0.05 mL of leaf sap was pressed out from each sample and put into the chamber of the potentiometer WP4C (*Decagon Devices*, Pullman, WA, the U.S.A.) to measure this parameter.

### Gas exchange measurements, chlorophyll fluorescence analysis and determination of the content of photosynthetic pigments

The net photosynthetic rate, transpiration rate and stomatal conductance were determined *in situ* using an LCi Portable Photosynthesis System (*ADC BioScientific*, Hoddesdon, the United Kingdom) with the following conditions in the measurement chamber: air temperature at 25°C, ambient CO_2_ concentration at 550±50 μL L^-1^, air flow rate at 205±30 μmol s^-1^, and irradiance at 300 μmol m^-2^ s^-1^ [25].

The polyphasic rise of the chlorophyll fluorescence transient (OJIP) was measured at the upper surface of the dark-adapted (20 min) leaves *in situ* with the portable fluorometer *FluorPen FP100max* (*Photon System Instruments*, Brno, Czech Republic) as described in [26]. The parameters of the JIP test (see S1 Table) were calculated according to the theory of energy flow in the photosynthetic electron-transport chain [29, 30]. The relative variable fluorescences W_OI_, W_OJ_, W_OK_ and W_IP_ (*i*.*e*., normalizations of the whole fluorescence transients) and the difference kinetics ΔW_OJ_ and ΔW_OK_ (as the differences between the drought-stressed and control plants) were also calculated according to [31] and their graphical representation was utilised to obtain further information on the primary photosynthetic processes.

The chlorophyll *a* and *b* contents and total carotenoids were determined spectrophotometrically [32] in the *N*,*N*-dimethylformamide extracts prepared as described in [33].

### The determination of various indicators of cell damage and protective mechanisms

Except for the CMI determination, all parameters were evaluated spectrophotometrically using *Anthelie Advanced 2* (*Secomam*, Alès, France) or *Evolution 201* (*Thermo Fisher Scientific*, Waltham, MA, the U.S.A.). The CMI was determined from the measurements of electrical conductivity of the samples consisting from 15 leaf discs with the *GRYF 158* conductometer (*Gryf HB*, Havlíčkův Brod, Czech Republic). The CMI was calculated as (100×C_1_/C_2_), where C_1_ is the electrical conductivity of a sample after 24 h incubation and C_2_ is the conductivity of the same sample after its denaturation. The method described in [34] was utilised for the determination of the content of MDA, resp. thiobarbituric acid reactive substances. This procedure serves as a method for the determination of a degree of membrane lipid peroxidation. The content of H_2_O_2_ was determined using the potassium iodide method described in [35]. The activities of APX and CAT in the leaves were assessed according to [36] and [37], for further details, see [26]. The protein content was determined by the Bradford method [38] using bovine serum albumin as a standard. The proline content was determined using the acid-ninhydrin method according to [39].

### Statistical analysis

The original data that constituted the basis for the statistical analysis are available in the S1 File. The datasets containing parameters that had to be measured on the same plants (see above) were subjected first to two-way analyses of variance (genotype, treatment (control/stress) and the interaction between these two factors were included as the sources of possible variability). To correct for false discovery rates (FDR), the Benjamini and Hochberg [40] correction was applied within the respective datasets with FDR set as 0.05. Tukey's HSD tests were then performed for each parameter separately. Additionally, to determine the relevant differences between the control and drought-stressed variants of the respective genotype, or the differences between both examined genotypes in plants either stressed or non-stressed by drought, one-way analyses of variance followed by Tukey's HSD tests were also performed. The statistical evaluation was conducted with CoStat (Version 6.204) statistical software (*CoHort Software*, Moterey, CA, the U.S.A.).

## Results

**Fig 1:**
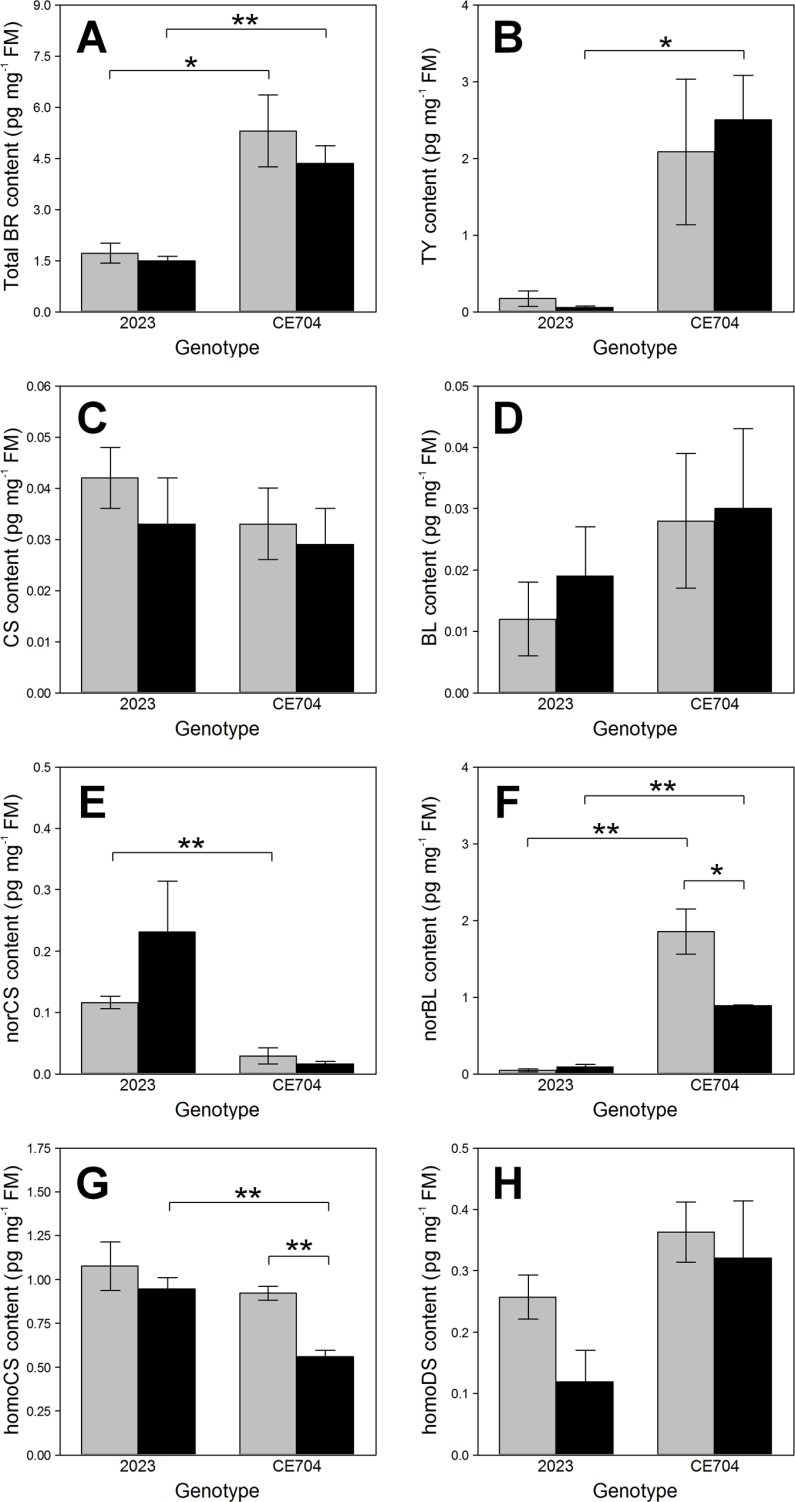
Contents of total and individual brassinosteroids (BRs) in two maize genotypes (2023 and CE704). Plants were either subjected to normal watering (control; grey columns) or to 14 days of withholding water (stress; black columns). Mean values ± SEM are shown (n = 3). Asterisks indicate significant (*p*≤0.05; *) or highly significant (*p*≤0.01; **) differences between mean values according to Tukey's tests made separately for each genotype (in case of the differences between control and stress treatment) or for each treatment (in case of the differences between both genotypes). BL … brassinolide, CS … castasterone, DS … dolichosterone, FM … leaf fresh mass, TY … typhasterol.

The contents and composition of BRs distinctly differed between both genotypes and in some cases also depended on water supply. CE704 displayed significantly higher levels of total BRs in its leaves compared to 2023 (Table in S2 File, Fig 1A). The most abundant BR detected in CE704 was TY followed by norBL. On the other hand, 2023 contained only small amounts of these two BRs per leaf fresh mass (FM) and the differences between both genotypes were statistically significant (Table in S2 File, Fig 1B and 1F). The CE704 stressed plants showed a significantly lower homoCS content in their leaves compared to 2023 (Table in S2 File, Fig 1G). The homoCS and homoDS contents were reduced by drought, particularly in 2023 (in case of the homoDS content non-significantly; Table in S2 File, Fig 1G and 1H). On the other hand, 2023 showed a distinct increase in the norCS content after drought, contrary to CE704. CE704 was generally characterised by a lower content of this BR (Table in S2 File, Fig 1E). Regarding two other BRs that were detected, *i*.*e*., CS and BL, no significant differences were observed between genotypes or between watering treatments (Table in S2 File, Fig 1C and 1D). All other analysed BRs (TE, norTE, *epi*BL, homoBL, *epi*CS, DL, homoDL, DS) were below a limit of a detection method.

**Fig 2:**
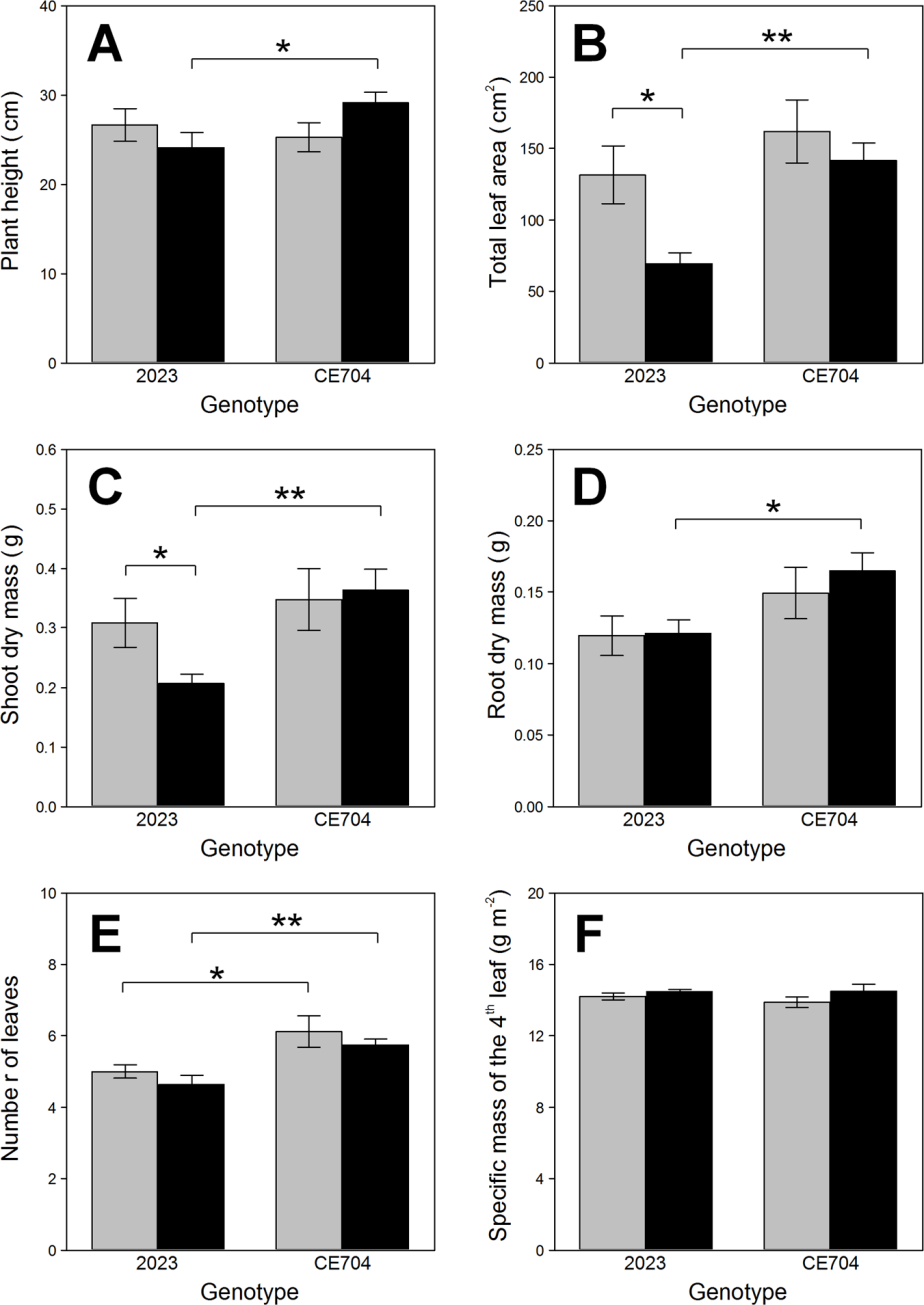
Selected parameters of plant morphology in two maize genotypes (2023 and CE704). Plants were either subjected to normal watering (control; grey columns) or to 14 days of withholding water (stress; black columns). Mean values ± SEM are shown (n = 8). Asterisks indicate significant (*p*≤0.05; *) or highly significant (*p*≤0.01; **) differences between mean values according to Tukey's tests made separately for each genotype (in case of the differences between control and stress treatment) or for each treatment (in case of the differences between both genotypes).

CE704 showed significantly higher values of plant height compared to 2023 but only under stress conditions (Fig 2A). This genotype also had higher number of visible leaves compared to 2023 (Table in S2 File, Fig 2E). While the older leaves of 2023 plants subjected to drought displayed strong symptoms of senescence (the first two leaves were completely dry or yellow, the third leaf also started to senesce), this did not apply to CE704, which developed almost normally (only slightly slowing down) even under stress conditions (S1 File, S1 Fig). Drought significantly reduced the total leaf area as well as the shoot dry mass in 2023 but not in CE704, resulting in genotypic differences under stress conditions (Table in S2 File, Fig 2B and 2C). The stressed plants of CE704 also had a greater root biomass compared to 2023; however, this did not apply to the control plants (Table in S2 File, Fig 2D). The SLM of the 4^th^ leaf did not significantly differ between both genotypes or between watering treatments (Table in S2 File, Fig 2F).

**Fig 3:**
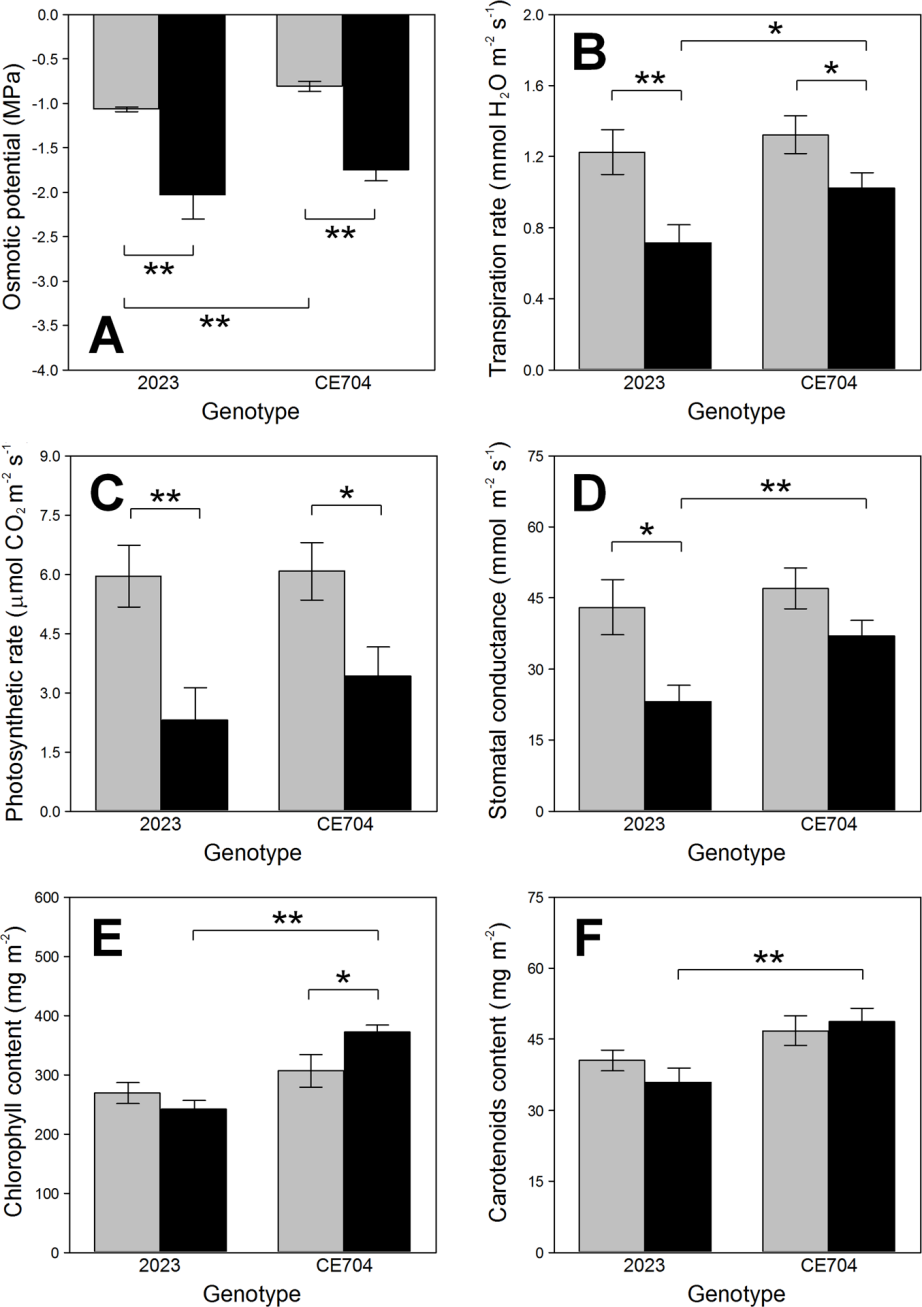
Selected parameters of gas exchange, the osmotic potential and the contents of chlorophylls and carotenoids in leaves of two maize genotypes (2023 and CE704). Plants were either subjected to normal watering (control; grey columns) or to 14 days of withholding water (stress; black columns). Mean values ± SEM are shown (n = 8 for gas exchange and the contents of photosynthetic pigments, n = 12 for osmotic potential). Asterisks indicate significant (*p*≤0.05; *) or highly significant (*p*≤0.01; **) differences between mean values according to Tukey's tests made separately for each genotype (in case of the differences between control and stress treatment) or for each treatment (in case of the differences between both genotypes).

The leaf osmotic potential values were lower in the drought-stressed plants of both genotypes compared to their respective controls (Table in S2 File, Fig 3A). However, the differences between the genotypes were statistically significant only for the control plants, not for the stressed ones (Fig 3A). The transpiration rate was reduced after 14 days without watering. This was less pronounced in CE704 than in 2023 (Fig 3B). Both the net photosynthetic rate and stomatal conductance were also negatively affected by drought and the changes in the values of these parameters were again more evident in 2023 than in CE704 (Table in S2 File, Fig 3C and 3D). This was reflected in the presence of significant differences between both genotypes in the stomatal conductance, which were found under stress conditions (Fig 3D).

The drought-stressed plants of CE704 contained more chlorophyll and carotenoids in its leaves compared to 2023 (Table in S2 File, Fig 3E and 3F). This was caused by the fact that the content of these chlorophylls actually increased in CE704 after 14 days without watering, contrary to 2023.

**Fig 4:**
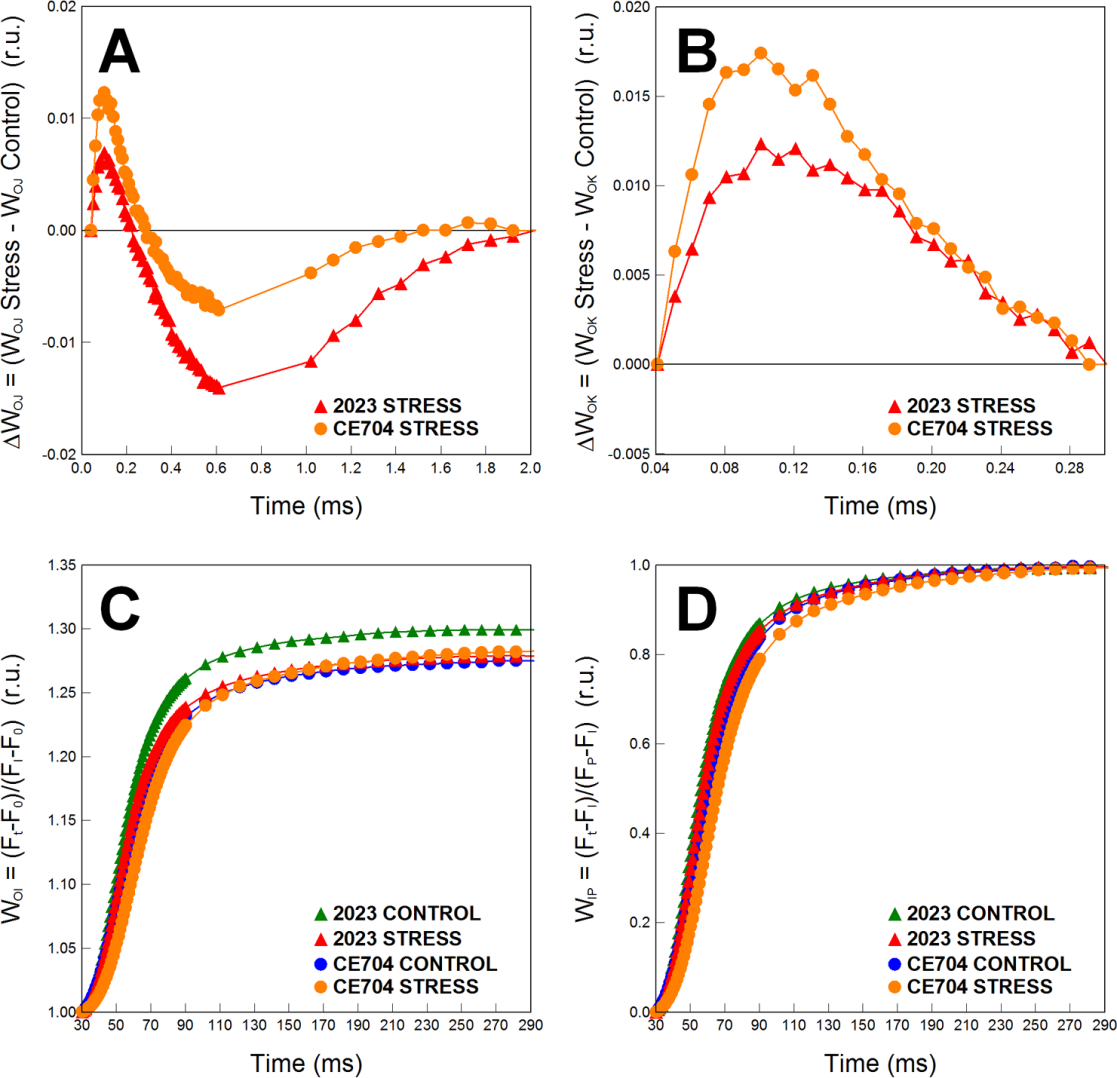
The difference kinetics and the relative variable fluorescences calculated from OJIP analysis of two maize genotypes (2023 and CE704). The difference kinetics ΔW_OJ_ (**A**) reveals the K-band; ΔW_OK_ (**B**) reveals the L-band. Only the part between the I and P points of the OJIP curve is shown for the relative variable fluorescence W_OI_ (**C**). The normalization of OJIP curve between the I and P points with the maximum amplitude fixed as 1 is shown as the relative variable fluorescence W_IP_ (**D**). Plants were subjected either to normal watering (control) or to 14 days of withholding water (stress). ΔW_OJ_ and ΔW_OK_ were calculated from the comparisons of the stressed and control plants; the latter are represented by the zero point of the respective y axes in graphs **A** and **B**. Mean values (n = 8) are shown. r.u. … relative units.

Drought also reduced the efficiency of the primary photosynthetic processes. There were apparent differences between the control and drought-stressed plants, particularly for the parameters describing electron transport within the photosystem (PS) II reaction centre, such as φ_P0_, φ_E0_ and ψ_E0_ as well as the performance index PI_ABS_, and these differences were more pronounced in the 2023 genotype (S1 File). However, no true statistically significant differences were found between the genotypes for the numerical parameters of the JIP test (Table in S2 File). The dissipation of excess excitation energy significantly increased with drought stress (parameters φ_E0_ and DI_0_/RC). The oxygen-evolving complex of PSII did not seem to be negatively affected in the drought-stressed plants of either genotype, as seen from the location of the K-band around zero on the graph of the ΔW_OJ_ difference kinetics (Fig 4A). The excitonic connectivity between the individual PSII units (inferred from the L-band positions above zero on the graph of the ΔW_OK_ difference kinetics) was slightly negatively affected by drought, but both genotypes did not differ in this respect (Fig 4B). Regarding the electron transport beyond PSII, our experimental plants were not affected much by drought (S1 File, Table in S2 File, parameters δ_RE01_, φ_RE01_ and ψ_RE01_). However, based on the greater observed difference in the positions of the respective W_OI_ curves, 2023 showed a slightly more pronounced decline in the reduction rate of electron acceptors at the end of the electron-transport chain due to drought than CE704 (Fig 4C). The size of the pool of these acceptors was similar in all the experimental variants (based on the fact that the positions of the W_IP_ curves did not differ much; Fig 4D).

**Fig 5:**
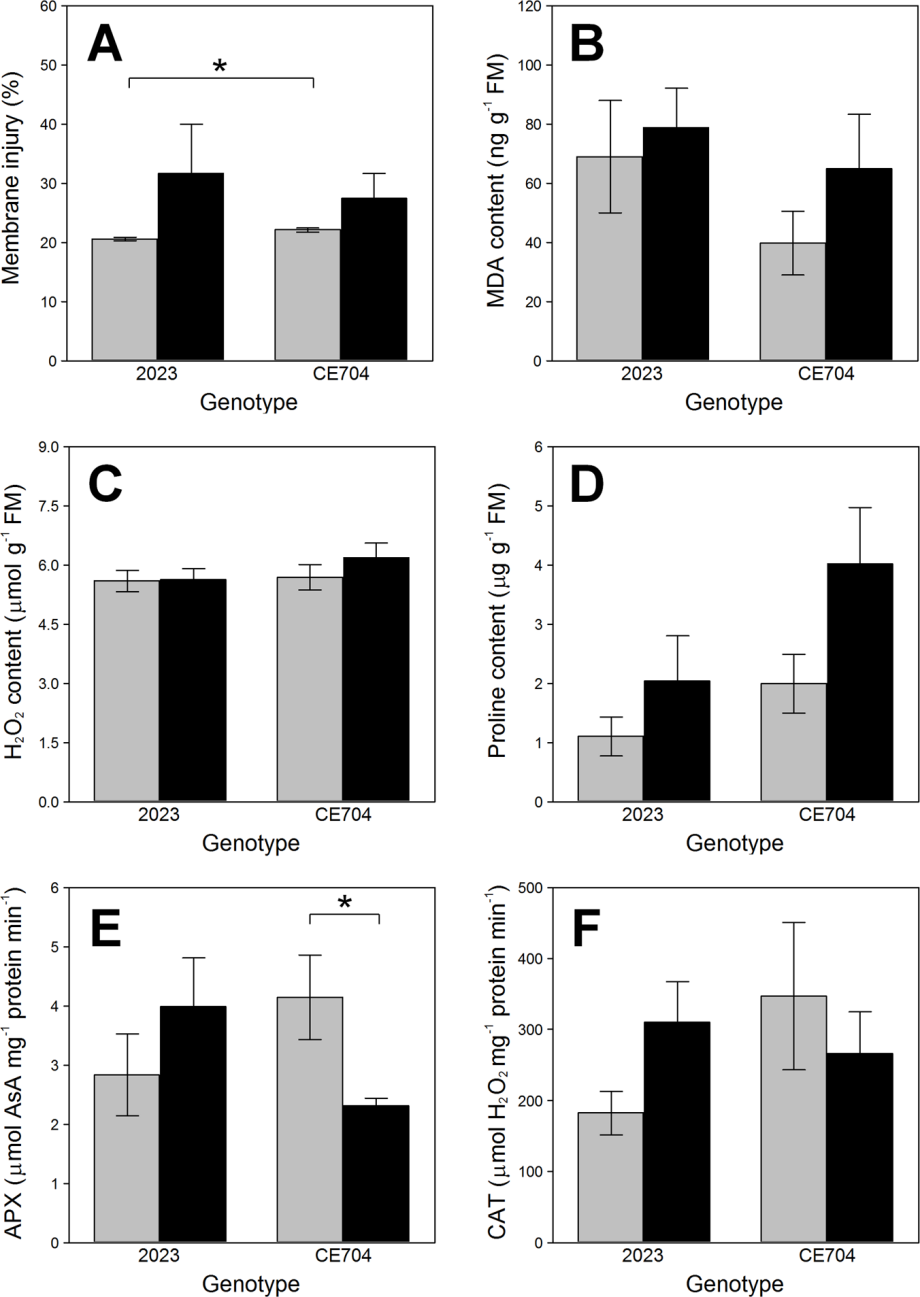
The contents of proline, H_2_O_2_, malondialdehyde (MDA), the antioxidant activities and the index of membrane injury in two maize genotypes (2023 and CE704). Plants were either subjected to normal watering (control; grey columns) or to 14 days of withholding water (stress; black columns). Mean values ± SEM are shown (n = 6 for the contents of MDA, n = 4 for the other parameters). Asterisks indicate significant (*p*≤0.05; *) or highly significant (*p*≤0.01; **) differences between mean values according to Tukey's tests made separately for each genotype (in case of the differences between control and stress treatment) or for each treatment (in case of the differences between both genotypes).AsA … ascorbate, APX … ascorbate peroxidase, CAT … catalase, FM … leaf fresh mass.

CMI had higher values in the stressed plants than in the control ones. This increase was not as prominent in CE704 as in 2023 (Fig 5A). The 2023 genotype generally also showed slightly higher peroxidation of membrane lipids based on the MDA content compared to CE704, although lipid peroxidation seemingly increased more in CE704 than in 2023 after the exposure of plants to drought (Fig 5B). CE704 was also characterised by a greater proline content in its leaves compared to 2023 and drought induced a further elevation of this osmoprotectant content in CE704 (Fig 5D). While the APX activity in the leaves of 2023 increased after 14 days without watering, the reverse was true for CE704 and a similar trend was observed for the CAT activity (Fig 5E and 5F). However, almost none of the described differences in the parameters characterising cell damage were actually statistically significant due to high variability in the samples (S1 File, Table in S2 File). No differences between genotypes or between control and stressed plants were found for the H_2_O_2_ content (Fig 5C).

## Discussion

Endogenous BRs are important regulators of various processes that occur in plants. Several studies have revealed that the contents of endogenous phytosterols (including campesterol and cholesterol which are the main precursors of BR biosynthesis) in plant leaves can significantly differ between genotypes [41, 42]. However, whether these differences also affect the levels of endogenous BRs has not been previously examined. To date, only Pociecha *et al*. reported the existence of genotypic differences in the response of CS levels in the leaves of two winter rye cultivars to cold acclimation [24].

The determination of various endogenous BRs in the leaves of our two maize genotypes with different drought sensitivities provided several interesting results. We found that the presence/content of the individual natural BRs in the leaves may depend on the genotype. The most abundant BR present in the leaves of our plants was TY (~2–3 pg mg^-1^ FM), followed by norBL (~1–2 pg mg^-1^ FM) and homoCS (~1 pg mg^-1^ FM). The drought-resistant CE704 genotype had particularly higher levels of both TY and norBL compared with the drought-sensitive genotype 2023. The presence of TY was reported previously for maize pollen [43] and the observed levels of TY in the leaves of our plants are mostly in agreement with those reported for the leaves or shoots of several other plant species [44]. However, the norBL content in the CE704 leaves reached 1–2 pg mg^-1^ FM, which might seem to be a rather high level for any BR detected in vegetative tissue, such as leaves [44], since these tissues generally contain significantly lower amounts of phytohormones compared with the reproductive tissues, such as pollen or seeds [45]. Indeed, the norBL level in our drought-resistant CE704 genotype is approximately 15 times higher compared to the norBL level reported for maize leaves by Oklešťková *et al*. [46]. This seems to be an interesting feature of this particular maize genotype. We are not aware of any previous study that reported the presence of this type of BR in such high quantity in this plant species or even its presence in any other monocot plant.

In addition to TY, norBL and homoCS, we also detected other endogenous BRs substances in smaller quantities, namely, homoDS, norCS, CS and BL. Although the biosynthetic pathways and mutual conversions of the C_27_, C_28_ and C_29_ BRs are not entirely clear at the present time, there is some evidence that the C_27_ BRs are formed from C_27_ sterol cholesterol while the C_29_ plant steroidal biosynthetic precursor for the production of C_29_ BRs is sitosterol [47]. In the C_28_ BRs group, TY is a precursor of CS which is converted into the biosynthetic end product BL. These reactions are probably unidirectional [48]. The conversion of CS to BL is catalysed by a cytochrome P-450 monooxygenase encoded by the *CYP85A2/3 (BR6ox2)* gene in the dicot plants *Arabidopsis* and tomato [49, 50]. BL plays an important role in the development of reproductive organs in these species, whereas CS probably serves as a regulator of plant vegetative growth [51]. Although BL has already been reported in rice shoots [52], other authors showed that the step of BR biosynthesis involving the conversion of CS to BL does not seem to exist in rice [53, 54]. The same situation seems to apply for other monocot grasses [55, 56]. However, BL was positively detected in leaves of graminaceous plants such as barley and wheat [57–59]. Therefore, the presence of BL and norBL in the leaves of our maize plants is perhaps not as exceptional if we also consider the lower detection limits of the method we used for their determination, compared with those used earlier by other authors. Clearly, monocot plants have either some means to perform the Baeyer-Villiger oxidation of CS to yield BL, or they can produce BL (and convert it to its various metabolites) by completely different reaction(s), although no details of this have been revealed.

Details of BR biosynthetic pathways were identified mostly in *Arabidopsis* and *Catharanthus roseus* [60]. Similar situation applies for BR signaling; an overwhelming majority of our knowledge of this topic comes from the model plant *Arabidopsis* [61]. Information on monocot plants is rather limited; although many aspects of BR biosynthesis and signaling seem to be conserved between dicots and monocots, some differences also exist [62]. Maize has not yet been much investigated in order to identify genes coding for proteins that catalyse individual steps of BR synthesis or participate in BR signaling pathways. To this date, only six such studies exist. The first discovery of a BR biosynthetic gene in maize was made by Tao *et al*. [63], who found a functional homolog of the *Arabidopsis DWF1* gene (*ZmDWF1*). Its product catalyses the reduction of 24-methylenecholesterol to campesterol at the beginning of the BR biosynthetic pathway and the gene was shown to be expressed particularly in roots. This agreed well with the subsequent studies of Kim *et al*. [64, 65], who demonstrated that BR biosynthesis in maize indeed occurs in roots. However, another study indicated that the *ZmDWF1* gene is expressed in all maize tissues (at least at the transcriptional level) with the highest degree of expression in the leaf collars and lower expression in older tissues [66]. Contrary to [63], the authors of this study also found not only one but two paralogs of *ZmDWF1* gene in the maize genome.

Another BR biosynthetic gene that was established in maize was *ZmDWF4* which codes for C-22 hydroxylase [67]. This rate-limiting enzyme catalyses various hydroxylation events at the beginning of both early and late C-6 oxidation pathways as well as the early C-22 oxidation pathway [60]. A higher level of its transcripts was found in shoots of young maize seedlings than in their roots. The third gene experimentally confirmed to participate in BR biosynthesis in this plant species was the maize homolog of *DET2*, expressed particularly in anthers [68]. Its product is 5α-steroid reductase, functioning mainly in the early C-22 oxidation and the late C-6 oxidation pathways [60]. The final BR biosynthetic gene documented to function in maize was revealed by [55]: *ZmBRD1*, an ortholog of the *BR6ox1* gene. The enzyme produced from this gene catalyses the final steps of early and late C-6 oxidation pathways, *i*.*e*. the synthesis of CS. Again, the highest expression of this gene was recorded in anthers but its transcripts were at least at some level present in all tissues. In addition to the four known maize BR biosynthetic genes, the gene coding for the BR receptor (*ZmBRI1*) was reported and experimentally confirmed by [69]. Although various additional orthologs of *Arabidopsis* genes known to participate in BR biosynthesis or signaling are present in the maize genome, their role in these processes has yet to be verified and it seems that at least some of them actually participate in other biological processes in this species (Tables in S3 File).

The organ-specific or developmental information on the expression of maize genes known/predicted to be involved in BR biosynthesis or signaling pathways can be easily found in the MaizeGDB (https://www.maizegdb.org/); it is based on the transcription data obtained by [70] and subsequently further updated [71]. Unfortunately, this does not give us any information on the possible changes of the expression of these genes that could be caused by unfavourable environmental conditions such as drought. Indeed, the relationship between BRs and drought at the molecular level is rather ill-defined. Recently, one group of scientists showed that some transcription factors from WRKY and NAC families, which are involved in plant stress responses, either directly interact with or influence the levels of some downstream components of the BR signaling pathway (particularly the transcription factor BES1) in *Arabidopsis* [72–75]. Based on their results, it seems that drought and BR pathways antagonize each other: BR signaling inhibits drought-induced gene expression pathways and *vice versa*. Some authors who worked with BR mutants, transgenic plants or plants treated with exogenous BRs and subjected to drought conditions, assessed the levels of a few specific transcripts presumed to be correlated to plant drought response. These studies were made with *Arabidopsis* [76], rapeseed [76, 77], cucumber [78], tobacco [79], potato [80], *Brachypodium* [81] or barley [82]. However, with the exception of Zhou *et al*., who found gradual increase in the levels of *CPD* transcripts during the first 24 hours of PEG-induced osmotic stress in potato (and subsequent drop to the original level after additional 24 hours) [80], the expression of BR biosynthetic or signaling genes in drought-stressed plants had not yet been purposefully analysed. The CPD protein catalyses some steps of the early C-22 oxidation and the late C-6 oxidation pathways [60].

We examined various differential gene expression studies available for drought-stressed maize (identified by searching the public databases NCBI GEO DataSets and GEO Profiles (https://www.ncbi.nlm.nih.gov/gds, https://www.ncbi.nlm.nih.gov/geoprofiles/) and the EMBL-EBI Expression Atlas (https://www.ebi.ac.uk/gxa/home) based on the Array Express database (https://www.ebi.ac.uk/arrayexpress/) and found some cases where the expression of maize genes known/predicted to be involved in BR biosynthesis and/or signaling (or maize orthologs of *Arabidopsis* BR genes) changed after the simulated drought (Table in S3 File). Generally, it seems that drought usually reduces the expression of BR biosynthetic genes at the transcriptional level; the maize orthologs of *Arabidopsis* BR biosynthetic genes, which showed elevated amounts of transcripts in drought-stressed plants, are probably involved more in the abscisic acid (ABA) or other metabolic pathways then directly in the BR biosynthesis (Tables in S3 File). However, the observed changes evidently depend on the analysed organ (*e*.*g*., [83–85]), its developmental stage [84, 85], the intensity/duration of stress [86–87] or a particular maize cultivar [86, 88]. The changes in the expression of genes predicted to be involved in BR signaling are even more variable (Tables in S3 File). Thus, no obvious conclusions on the relationship between the transcription of these genes and drought can be drawn, particularly as true participation of the majority of these genes in BR-signaling pathways of maize has yet to be confirmed.

Moreover, although transcriptomic analyses are rather popular, they can give only a limited picture of gene expression changes. Drought-induced changes of plant cell transcriptome do not have to be reflected in the changes of cell proteome (due to many steps and factors influencing gene expression between RNA and protein levels). Indeed, among studies dealing with drought-induced changes in plant proteome, that also simultaneously analysed the changes of transcriptome [89–96], all (with the exception of [93]) reported that no true correlation between these two sets of gene expression data existed. Our previous analyses of drought-induced changes of leaf proteome performed in the same two maize genotypes we used in our present study did not reveal any significant change in the amounts of any protein known or predicted to be involved in BR biosynthesis or signaling [25, 26]. Nolan *et al*. [97], who examined the regulation of *BES1* expression in *Arabidopsis* plants exposed to drought conditions, found that although no change in *BES1* transcript levels was observed in stressed plants, the levels of the BES1 protein were reduced after drought exposure. Thus, the expression of this component of BR-signaling pathway was regulated at the posttranscriptional level and probably even at the posttranslational level (the stability of the BES1 protein). Lei *et al*. [98] performed a ribosome profiling assay together with a RNAseq-based transcriptome analysis in maize seedlings grown in normal and drought conditions. Several genes involved in BR biosynthesis (*e*.*g*., *ZmDWF1* and an ortholog of *Arabidopsis DWF7*) or signaling (*e*.*g*., *ZmBRI1* and orthologs of *Arabidopsis BES1*, *BRH1*, *BEH3/BEH4*) showed reduced translational efficiency but no changes at the level of their mRNAs [98]. Clearly, the relationships between the transcriptional, translational and post-translational levels of expression of BR-associated genes in plants exposed to drought and their influence on the contents of endogenous BRs and general plant response to this stress factor can be very complex and deserve further examination.

Our experiments with drought-stressed maize plants revealed several changes in the content of endogenous BRs induced by this stress factor. The most conspicuous change was the significant reduction of the homoCS and norBL levels in the drought-resistant CE704 genotype. This differs from the results of Gruszka *et al*. [23], who did not observe any particular drought-induced reduction in the homoCS content in the leaves of their barley plants. On the other hand, they reported that drought specifically induces *epi*BL formation. Interestingly, this drought-specific elevation of the *epi*BL content did not depend on functional BR biosynthesis or signalling, because it was observed in BR mutants as well as in *wild type* plants. They also found rather high CS content that increased with drought, which also does not agree with our observations, as the levels of CS in the leaves of our plants did not change with water deficiency. The difference between our results regarding the changes in the CS content and its derivatives and the results of Gruszka *et al*. [23] could perhaps arise from the evolutionary divergence of the *BR6ox* gene between barley and maize species. Maize has only one homologue of the *BR6ox* gene [55], whereas the barley genome contains two homologs of this gene (*HvDWARF* and *HvBRD*), which are probably partly redundant at least in CS biosynthesis [99]. Whether one of these proteins could still have another function under specific (drought) conditions remains to be seen.

Another possible explanation for the absence of any significant changes of the CS content in our experimental plants could lie in the mutual conversions of the C_27_, C_28_ and C_29_ BRs. CS can be converted to norCS and, likewise, norBL can be produced from BL. Both these norBRs belong to the C_27_ group and their biosynthesis in *Arabidopsis* and tomato originates from cholesterol [100–103]. However, norCS can also be methylated to yield CS, with DS acting as an intermediate. Reportedly, this conversion occurs also in monocot plants [47, 65]. Furthermore, CS can also be produced from C_29_ BRs such as homoCS or homoDS in rice [47]. Both homoBRs mentioned were also detected at a certain quantity in our maize samples; thus, we dare to say that this pathway operates in maize as another representative of monocot plants.

As the leaves of our drought-sensitive 2023 inbred line showed an elevation of the norCS content whereas the homoDS content was reduced, it is possible that the conversions between these BRs masked an actual inducement of CS biosynthesis by drought in this genotype. Jäger *et al*. [22], who described an increase in the CS content after pea plant exposure to a water deficit, proposed that the induction of CS levels could be correlated with a large enhancement of the ABA content, which occurs under conditions of *severe* drought stress. This applied for their experiment, as well as to the abovementioned experiments of Gruszka *et al*. [23], who also observed a substantial increase in the levels of ABA in their drought-exposed barley. The drought-sensitive 2023 clearly suffered from drought more than CE704, as evident, from the more pronounced reduction of its biomass and photosynthetic efficiency, and suggested by some other parameters correlated to cell damage and antioxidative protection. It could be thus experiencing symptoms of more severe drought stress, contrary to CE704. The different degree of drought sensitivity of both genotypes could also explain why we did not find similar changes in the norCS and homoDS contents in the CE704 genotype, because this genotype was much less stressed by water deficiency.

Our CE704 genotype has one interesting trait: it can keep the stomata opened (at least to some degree) when watering ceases. We have previously shown [25, 26] that this ability enables it to maintain a sufficiently high photosynthetic rate during the *early* stage of drought response, which results in the unimpeded production of the energetically rich compounds necessary for proteosynthesis. This early acclimation mechanism, together with some other aspects of CE704 physiology and biochemistry (regarding, *e*.*g*., the higher proline content), enables it to better counteract the negative consequences of drought stress during the later, more severe phases [25, 26]. BRs are known to indirectly regulate stomatal function in a concentration- and species-dependent manner [104, 105]. While low concentrations of exogenously applied BRs do not affect stomatal movements, higher BR concentrations can operate in two opposite ways. They either induce stomatal closure [105, 106] or, conversely, facilitate the opening of the stomata whose closure was induced by ABA [107] or impaired by BR deficiency [108]. It is thus possible that the observed differences in the endogenous BR levels in the leaves of our two maize genotypes could perhaps be somehow connected to the behaviour of their stomata. A further analysis of this phenomenon is certainly needed.

## Conclusions

This is the first study investigating the endogenous BR content in maize genotypes differing in their drought sensitivity. We found that TY together with norBL and homoCS are the main representatives of this group of phytohormones in maize leaves. Importantly, this is also the first report of presence of a rather high quantity of norBL in a monocot plant. We also revealed for the first time that drought-resistant and drought-sensitive maize genotypes differ in the presence/contents of individual, naturally-occurring BRs. The observed differences between both genotypes in the endogenous BR content are probably correlated with their different degrees of drought sensitivity, which was demonstrated at the levels of plant morphology, physiology and biochemistry. We confirmed our original hypothesis that the drought-resistant genotype displays higher levels of endogenous BRs compared with the sensitive genotype already under non-stress conditions. Indeed, this could be one of the reasons for its higher drought resistance. Our second hypothesis suggested that the resistant genotype should not need to elevate its endogenous BR contents when subjected to water deficiency, because it would not experience stress to such a degree as the drought-sensitive genotype, which was also supported by our data.

## Supporting information

S1 Table Selected photosynthetic parameters of the JIP test derived from the measurements of the polyphasic rise of chlorophyll *a* fluorescence. F_0_—the initial fluorescence intensity (at 50 μs), F_K_—the fluorescence intensity at the K-step (300 μs), F_J_—the fluorescence intensity at the J-step (at 2 ms), F_I_—the fluorescence intensity at the I-step (at 30 ms), F_M_ ≈ F_P_—the maximum fluorescence intensity, Area—area between the fluorescence curve and F_M_, PSI—photosystem I, PSII—photosystem II, RC—reaction centre.
(DOCX)

S1 File The original data on the brassinosteroid contents, plant morphology and various physiological and biochemical characteristics measured in two maize genotypes (2023 and CE704). Plants were either subjected to normal watering (control) or to 14 days of withholding water (stress).
(XLSX)

S2 File Tables with the results of the two-way ANOVA and Tukey's tests applied to various physiological and biochemical parameters measured in two maize genotypes (2023 and CE704). Plants were either subjected to normal watering (control) or to 14 days of withholding water (stress).
(DOCX)

S3 File Tables with the main results and experimental aspects of the transcriptome studies performed with drought-stressed maize, which showed differential expression of genes involved in brassinosteroid biosynthesis, catabolism/homeostasis or signaling. (DOCX)

S1 Fig **Phenotypic representation of two maize genotypes, 2023 (A, B) and CE704 (C, D).** Plants were subjected either to normal watering (control; **A, C**) or to 14 days of withholding water (stress; **B, D**).
(TIF)
