# Supplementary material for: Drought-tolerant and drought-sensitive genotypes of maize (Zea mays L.) differ in contents of endogenous brassinosteroids and their drought-induced changes
Source: PLoS One. 2018 May 24;13(5):e0197870. doi: 10.1371/journal.pone.0197870 (PMC5967837; doi:10.1371/journal.pone.0197870)
Supplement: S1 Table — F0—the initial fluorescence intensity (at 50 μs), FK—the fluorescence intensity at the K-step (300 μs), FJ—the fluorescence intensity at the J-step (at 2 ms), FI—the fluorescence intensity at the I-step (at 30 ms), FM ≈ FP—the maximum fluorescence intensity, Area—area between the fluorescence curve and FM, PSI—photosystem I, PSII—photosystem II, RC—reaction centre. (DOCX) [file pone.0197870.s001.docx]

**Supplementary Table 1** Selected photosynthetic parameters of the JIP test derived from the measurements of the polyphasic rise of chlorophyll *a* fluorescence. F_0_ - the initial fluorescence intensity (at 50 µs), F_K_ - the fluorescence intensity at the K-step (300 µs), F_J_ - the fluorescence intensity at the J-step (at 2 ms), F_I_ - the fluorescence intensity at the I-step (at 30 ms), F_M_ ≈ F_P_ - the maximum fluorescence intensity, Area - area between the fluorescence curve and F_M_, PSI - photosystem I, PSII - photosystem II, RC - reaction centre.

| Parameter | Definition | Formula |
| --- | --- | --- |
| V_J_ | Relative variable fluorescence at the J-step | (F_J_-F_0_)/(F_M_-F_0_) |
| V_I_ | Relative variable fluorescence at the I-step | (F_I_-F_0_)/(F_M_-F_0_) |
| M_0_ | Approximated initial slope of the fluorescence transient | 4(F_K_-F_0_)/(F_M_-F_0_) |
| S_M_ | Normalized total complementary area (reflecting multiple-turnover Q_A_ reduction events) | Area/(F_M_-F_0_) |
| S_S_ | Normalized total complementary area corresponding to O-J phase (reflecting single-turnover Q_A_ reduction events) | V_J_/M_0_ |
| φ_P0_ | Maximum quantum yield of primary PSII photochemistry | (F_M_-F_0_)/F_M_ |
| φ_E0_ | Quantum yield of electron transport flux from Q_A_ to Q_B_ | [1-(F_0_/F_M_)](1-V_J_) |
| φ_RE0_ | Quantum yield of electron transport flux until the PSI electron acceptors | 1-(F_I_/F_M_) |
| φ_D0_ | Quantum yield of energy dissipation | F_0_/F_M_ |
| ψ_E0_ | Efficiency/probability with which a PSII trapped electron is transferred from Q_A_ to Q_B_ | 1-V_J_ |
| ψ_RE0_ | Efficiency/probability with which a PSII trapped electron is transferred until PSI acceptors | 1-V_I_ |
| δ_RE0_ | Efficiency/probability with which an electron from Q_B_ is transferred until PSI acceptors | (1-V_I_)/(1-V_J_) |
| γ_RC_ | Probability that a PSII chlorophyll functions as RC | 1/(ABS/RC+1) |
| ABS/RC | Average absorbed photon flux per PSII RC (apparent antenna size of an active PSII) | (M_0_/V_J_)(1/φ_P0_) |
| TP_0_/RC | Maximum trapped exciton flux per PSII | M_0_/V_J_ |
| ET_0_/RC | Electron transport flux from Q_A_ to Q_B_ per PSII | (M_0_/V_J_)ψ_E0_ |
| RE_0_/RC | Electron transport flux until PSI acceptors per PSII | (M_0_/V_J_)ψ_RE0_ |
| DI_0_/RC | Dissipated energy flux per PSII | (ABS/RC)-(TP_0_/RC) |
| PI_ABS_ | Performance index for the energy conservation from photons absorbed by PSII antenna, to the reduction of Q_B_ | [1/(ABS/RC)][φ_P0_/(1-φ_P0_)][ψ_E0_/(1-ψ_E0_)] |
| PI_TOTAL_ | Performance index for the energy conservation from photons absorbed by PSII antenna, until the reduction of PSI acceptors | PI_ABS_[δ_RE0_/(1-δ_RE0_)] |
