# Supplementary material for: Drought-tolerant and drought-sensitive genotypes of maize (Zea mays L.) differ in contents of endogenous brassinosteroids and their drought-induced changes
Source: PLoS One. 2018 May 24;13(5):e0197870. doi: 10.1371/journal.pone.0197870 (PMC5967837; doi:10.1371/journal.pone.0197870)
Supplement: S2 File — Plants were either subjected to normal watering (control) or to 14 days of withholding water (stress). (DOCX) [file pone.0197870.s003.docx]

**Results of the two-way ANOVA and Tukey´s tests applied to brassinosteroid contents measured in leaves of two maize genotypes (2023 and CE704).**

| **Parameter** | **Two-way ANOVA** | | | **Tukey´s test** | |
| --- | --- | --- | --- | --- | --- |
|  | **Genotype** | **Treatment** | **G×T** | **Genotype** | **Treatment** |
| Total brassinosteroids | 0.004 | 0.544 | 0.806 | 2023<CE704 | Control=Stress |
| Typhasterol | 0.013 | 0.794 | 0.806 | 2023<CE704 | Control=Stress |
| Castasterone | 0.386 | 0.544 | 0.806 | 2023=CE704 | Control=Stress |
| Brassinolide | 0.226 | 0.754 | 0.806 | 2023=CE704 | Control=Stress |
| 28-norcastasterone | 0.014 | 0.532 | 0.509 | 2023>CE704 | Control=Stress |
| 28-norbrassinolide | 0.000 | 0.064 | 0.072 | 2023<CE704 | Control>Stress |
| 28-homocastasterone | 0.018 | 0.064 | 0.509 | 2023>CE704 | Control>Stress |
| 28-homodolichosterone | 0.048 | 0.477 | 0.806 | 2023<CE704 | Control=Stress |

Plants were either subjected to normal watering (Control) or to 14 days of withholding water (Stress). The significance (p) levels adjusted using Benjamini Hochberg False Discovery Rate (FDR) adjustment (FDR=0.05) are shown for the factors included in the analysis: the differences between genotypes, the differences between control and stress treatments and the interaction between these two factors (G×T). The equal sign means that no significant differences were found between the respective genotypes/treatments, signs > and < mean that the respective genotype/treatment showed significantly higher/lower values compared to the other one according to the Tukey´s test (p≤0.05).

**Results of the two-way ANOVA and Tukey´s tests applied to various morphological parameters measured in two maize genotypes (2023 and CE704).**

| **Parameter** | **Two-way ANOVA** | | | **Tukey´s test** | |
| --- | --- | --- | --- | --- | --- |
|  | **Genotype** | **Treatment** | **G×T** | **Genotype** | **Treatment** |
| Plant height | 0.364 | 0.802 | 0.235 | 2023=CE704 | Control=Stress |
| Total leaf area | 0.028 | 0.175 | 0.499 | 2023<CE704 | Control>Stress |
| Total shoot dry mass | 0.053 | 0.448 | 0.384 | 2023<CE704 | Control=Stress |
| Total root dry mass | 0.053 | 0.735 | 0.862 | 2023<CE704 | Control=Stress |
| Number of leaves | 0.014 | 0.448 | 1.000 | 2023<CE704 | Control=Stress |
| Area of the 1^st^ leaf | 0.684 | 0.448 | 0.235 | 2023=CE704 | Control=Stress |
| Area of the 2^nd^ leaf | 0.274 | 0.175 | 0.878 | 2023=CE704 | Control>Stress |
| Area of the 3^rd^ leaf | 0.093 | 0.971 | 0.896 | 2023>CE704 | Control=Stress |
| Area of the 4^th^ leaf | 0.382 | 0.392 | 0.042 | 2023=CE704 | Control=Stress |
| Dry mass of the 1^st^ leaf | 0.118 | 0.802 | 0.588 | 2023=CE704 | Control=Stress |
| Dry mass of the 2^nd^ leaf | 0.126 | 0.448 | 0.522 | 2023=CE704 | Control=Stress |
| Dry mass of the 3^rd^ leaf | 0.053 | 0.971 | 0.896 | 2023>CE704 | Control=Stress |
| Dry mass of the 4^th^ leaf | 0.684 | 0.448 | 0.105 | 2023=CE704 | Control=Stress |
| Specific mass of the 4^th^ leaf | 0.681 | 0.410 | 0.768 | 2023=CE704 | Control=Stress |

Plants were either subjected to normal watering (Control) or to 14 days of withholding water (Stress). The significance (p) levels adjusted using Benjamini Hochberg False Discovery Rate (FDR) adjustment (FDR=0.05) are shown for the factors included in the analysis: the differences between genotypes, the differences between control and stress treatments and the interaction between these two factors (G×T). The equal sign means that no significant differences were found between the respective genotypes/treatments, signs > and < mean that the respective genotype/treatment showed significantly higher/lower values compared to the other one according to the Tukey´s test (p≤0.05).

**Results of the two-way ANOVA and Tukey´s tests applied to gas exchange parameters and osmotic potential measured in leaves of two maize genotypes (2023 and CE704).**

| **Parameter** | **Two-way ANOVA** | | | **Tukey´s test** | |
| --- | --- | --- | --- | --- | --- |
|  | **Genotype** | **Treatment** | **G×T** | **Genotype** | **Treatment** |
| Leaf osmotic potential | 0.108 | 0 | 0.672 | 2023=CE704 | Control>Stress |
| Transpiration rate | 0.108 | 0.001 | 0.672 | 2023=CE704 | Control>Stress |
| Stomatal conductance | 0.108 | 0.002 | 0.695 | 2023<CE704 | Control>Stress |
| Net photosynthetic rate | 0.424 | 0 | 0.937 | 2023=CE704 | Control>Stress |

Plants were either subjected to normal watering (Control) or to 14 days of withholding water (Stress). The significance (p) levels adjusted using Benjamini Hochberg False Discovery Rate (FDR) adjustment (FDR=0.05) are shown for the factors included in the analysis: the differences between genotypes, the differences between control and stress treatments and the interaction between these two factors (G×T). The equal sign means that no significant differences were found between the respective genotypes/treatments, signs > and < mean that the respective genotype/treatment showed significantly higher/lower values compared to the other one according to the Tukey´s test (p≤0.05).

**Results of the two-way ANOVA and Tukey´s tests applied to the content of photosynthetic pigments and selected photosynthetic parameters of the JIP test measured in leaves of two maize genotypes (2023 and CE704).**

| **Parameter** | **Two-way ANOVA** | | | **Tukey´s test** | |
| --- | --- | --- | --- | --- | --- |
|  | **Genotype** | **Treatment** | **G×T** | **Genotype** | **Treatment** |
| Chlorophyll *a* content | 0.000 | 0.501 | 0.161 | 2023<CE704 | Control=Stress |
| Chlorophyll *b* content | 0.000 | 0.202 | 0.161 | 2023<CE704 | Control=Stress |
| Total chlorophyll content | 0.000 | 0.418 | 0.161 | 2023<CE704 | Control=Stress |
| Total carotenoids content | 0.011 | 0.665 | 0.421 | 2023<CE704 | Control=Stress |
| V_J_ | 0.948 | 0.007 | 0.321 | 2023=CE704 | Control<Stress |
| V_I_ | 0.430 | 0.563 | 0.367 | 2023=CE704 | Control=Stress |
| M_0_ | 0.430 | 0.044 | 0.409 | 2023=CE704 | Control<Stress |
| φ_P0_ | 0.128 | 0.006 | 0.729 | 2023<CE704 | Control>Stress |
| φ_E0_ | 0.572 | 0.006 | 0.421 | 2023=CE704 | Control>Stress |
| φ_RE01_ | 0.400 | 0.292 | 0.421 | 2023=CE704 | Control=Stress |
| φ_D0_ | 0.128 | 0.006 | 0.729 | 2023<CE704 | Control<Stress |
| ψ_E0_ | 0.948 | 0.007 | 0.321 | 2023=CE704 | Control>Stress |
| ψ_RE01_ | 0.430 | 0.563 | 0.367 | 2023=CE704 | Control=Stress |
| δ_RE01_ | 0.430 | 0.271 | 0.731 | 2023=CE704 | Control=Stress |
| γRC | 0.766 | 0.292 | 0.797 | 2023=CE704 | Control=Stress |
| ABS/RC | 0.766 | 0.292 | 0.781 | 2023=CE704 | Control=Stress |
| TP_0_/RC | 0.364 | 0.846 | 0.891 | 2023=CE704 | Control=Stress |
| ET_0_/RC | 0.430 | 0.030 | 0.367 | 2023=CE704 | Control>Stress |
| RE_01_/RC | 0.317 | 0.563 | 0.367 | 2023=CE704 | Control=Stress |
| DI_0_/RC | 0.430 | 0.016 | 0.729 | 2023=CE704 | Control<Stress |
| PI_ABS_ | 0.430 | 0.006 | 0.731 | 2023=CE704 | Control>Stress |
| PI_TOTAL_ | 0.364 | 0.064 | 0.729 | 2023=CE704 | Control>Stress |

Plants were either subjected to normal watering (Control) or to 14 days of withholding water (Stress). The significance (p) levels adjusted using Benjamini Hochberg False Discovery Rate (FDR) adjustment (FDR=0.05) are shown for the factors included in the analysis: the differences between genotypes, the differences between control and stress treatments and the interaction between these two factors (G×T). The equal sign means that no significant differences were found between the respective genotypes/treatments, signs > and < mean that the respective genotype/treatment showed significantly higher/lower values compared to the other one according to the Tukey´s test (p≤0.05).

**Results of the two-way ANOVA and Tukey´s tests applied to various indicators of cell damage and protective mechanisms measured in leaves of two maize genotypes (2023 and CE704).**

| **Parameter** | **Two-way ANOVA** | | | **Tukey´s test** | |
| --- | --- | --- | --- | --- | --- |
|  | **Genotype** | **Treatment** | **G×T** | **Genotype** | **Treatment** |
| Cell membrane injury index | 0.800 | 0.351 | 0.631 | 2023=CE704 | Control=Stress |
| Malondialdehyde content | 0.564 | 0.562 | 0.631 | 2023=CE704 | Control=Stress |
| H_2_O_2_ content | 0.587 | 0.618 | 0.631 | 2023=CE704 | Control=Stress |
| Ascorbate peroxidase activity | 0.800 | 0.731 | 0.240 | 2023=CE704 | Control=Stress |
| Catalase activity | 0.587 | 0.740 | 0.453 | 2023=CE704 | Control=Stress |
| Proline content | 0.330 | 0.294 | 0.631 | 2023=CE704 | Control<Stress |

Plants were either subjected to normal watering (Control) or to 14 days of withholding water (Stress). The significance (p) levels adjusted using Benjamini Hochberg False Discovery Rate (FDR) adjustment (FDR=0.05) are shown for the factors included in the analysis: the differences between genotypes, the differences between control and stress treatments and the interaction between these two factors (G×T). The equal sign means that no significant differences were found between the respective genotypes/treatments, signs > and < mean that the respective genotype/treatment showed significantly higher/lower values compared to the other one according to the Tukey´s test (p≤0.05).
