# Supplementary material for: Drought-tolerant and drought-sensitive genotypes of maize (Zea mays L.) differ in contents of endogenous brassinosteroids and their drought-induced changes
Source: PLoS One. 2018 May 24;13(5):e0197870. doi: 10.1371/journal.pone.0197870 (PMC5967837; doi:10.1371/journal.pone.0197870)
Supplement: S3 File — (DOCX) [file pone.0197870.s004.docx]

**Maize genes known/predicted to be involved in brassinosteroid biosynthesis and/or catabolism/homeostasis.** The gene list was prepared using the NCBI Gene database (https://www.ncbi.nlm.nih.gov/gene/), the Gramene database (http://www.gramene.org/) and the MaizeGDB database (https://www.maizegdb.org/). Genes that were experimentally verified to participate in brassinosteroid biosynthesis in this species (the respective references are given in the main text of the paper) are shown in bold. Additionally, maize orthologs to *Arabidopsis thaliana* genes known to be involved in brassinosteroid biosynthesis according to the TAIR database (https://www.arabidopsis.org/) are also included; these are shown in italics (*Arabidopsis* genes with no known orthologs in the maize genome are listed below the table). Gene orthology was determined using the Putative Orthologous Groups database (http://cas-pogs.uoregon.edu/#/).

| **Gene ID/synonym(s)** | **Gene/protein description** | **Orthologous gene(s) in *A. thaliana*** |
| --- | --- | --- |
| *Zm00001d002629, GRMZM2G096086, 103646058* | *cytochrome P450 87A3, putative cytochrome P450 superfamily protein* | *AT1G12740, CYP87A2* |
| Zm00001d003349, GRMZM2G107199, 100217085 | uncharacterized protein LOC100217085, cytochrome P450 CYP724B3, putative cytochrome P450 superfamily protein | AT5G14400, CYP724A1 |
| Zm00001d004957, GRMZM2G162737, *103647105* | cytochrome P450 90A4, cytochrome P450 90A1, cytochrome P450 CYP90A22v2 | AT5G05690, CPD/CBB3/DWF3/CYP90A |
| *Zm00001d005889, GRMZM2G002142, 103647484* | *abscisic acid 8'-hydroxylase 3, putative cytochrome P450 superfamily protein* | *AT3G19270, CYP707A4* |
| *Zm00001d008569, GRMZM2G167085, 100282855* | *delta-7-sterol-C5, delta(7)-sterol-C5(6)-desaturase 1* | *AT3G02580, STE1/BUL1/DWF7* |
| Zm00001d011029 | 6-deoxotyphasterol C-23 hydroxylase |  |
| *Zm00001d011117, GRMZM2G363429, 100501664* | *putative cytochrome P450 superfamily protein, cytochrome P450 family 722 subfamily A polypeptide 1* | *AT1G19630, CYP722A1* |
| *Zm00001d013629, GRMZM2G100412, 103626026* | *cytochrome P450 87A3, cytochrome P450 family 87 subfamily A polypeptide 2, putative cytochrome P450 superfamily protein* | *AT1G12740, CYP87A2* |
| *Zm00001d013720, GRMZM2G446454, 109939182* | *cytochrome P450 87A3, cytochrome P450 family 87 subfamily A polypeptide 2* | *AT1G12740, CYP87A2* |
| *Zm00001d015721, GRMZM2G045319, 103626790* | *cytochrome P450 734A2, Cytochrome P450 734A1, putative cytochrome P450 superfamily protein* | *AT2G26710, BAS1/CYP72B1* |
| **Zm00001d014887, GRMZM2G057000, DWF1, 542549** | **nana plant2, 24-methylenechloesterol reductase, DWF1 like1, steroid 24 isomerase/reductase** | **AT3G19820, DWF1/CBB1/DIM** |
| Zm00001d016463, GRMZM2G027886, 100282098, pco110886 | uncharacterized protein LOC100282098, pco110886(425), 7-dehydrocholesterol reductase | AT1G50430, DWF5 |
| *Zm00001d017582, GRMZM2G035309, 103627541* | *cytochrome P450 87A3, cytochrome P450 family 87 subfamily A polypeptide 2, putative cytochrome P450 superfamily protein* | *AT1G12740, CYP87A2* |
| *Zm00001d017762, GRMZM2G179147, 100383693* | *putative cytochrome P450 superfamily protein, abscisic acid 8'-hydroxylase1* | *AT2G29090, CYP707A2; AT4G19230, CYP707A1; AT5G45340, CYP707A3* |
| *Zm00001d020717, GRMZM2G065928, 100191697* | *uncharacterized protein LOC100191697, putative cytochrome P450 superfamily protein, putative cytochrome P450 superfamily protein* | *AT3G19270, CYP707A4* |
| Zm00001d020730 | (5α)-campestan-3-one hydroxylase, campestanol hydroxylase, campest-4-en-3-one hydroxylase |  |
| *Zm00001d022351, GRMZM2G019666, 103634425* | *cytochrome P450 734A5, cytochrome P450 734A1, putative cytochrome P450 superfamily protein* | *AT2G26710, BAS1/CYP72B1* |
| Zm00001d026008 | 6-deoxotyphasterol C-23 hydroxylase |  |
| *Zm00001d026083, GRMZM2G702529, 103641956* | *cytochrome P450 87A3, putative cytochrome P450 superfamily protein* | *AT1G12740, CYP87A2* |
| Zm00001d027497, GRMZM2G137380, 103644399 | 6-deoxotyphasterol C-23 hydroxylase, uncharacterized LOC103644399, putative HLH DNA-binding domain superfamily protein |  |
| **Zm00001d028325, GRMZM2G065635, DWF4, 100191562** | **putative cytochrome P450 superfamily protein, brassinosteroid synthesis1, cytochrome P450 CYP90B14v1, cytochrome P450 CYP90B14v2, steroid 22 α hydroxylase protein** | **AT3G50660, DWF4** |
| **Zm00001d033180, GRMZM2G103773, CYP85A1, 100193331** | **putative cytochrome P450 superfamily protein, brassinosteroid-deficient dwarf1** | **AT3G30180, BR6OX2/CYP85A2;**  **AT5G38970, BR6OX1/CYP85A1/BRD1** |
| *Zm00001d033470, GRMZM2G303426* | *cytochrome P450 family 87 subfamily A polypeptide 2* | *AT1G12740, CYP87A2* |
| Zm00001d037745, GRMZM2G032896, 103630138 | cytochrome P450 90D2, cytochrome P450 CYP90D10.b, putative cytochrome P450 superfamily protein | AT3G13730, CYP90D1 |
| Zm00001d038967 | 6-deoxotyphasterol C-23 hydroxylase |  |
| Zm00001d039453, GRMZM2G143235, 100384722 | putative cytochrome P450 superfamily protein, 3-epi-6-deoxocathasterone 23-monooxygenase | AT3G13730, CYP90D1 |
| *Zm00001d039650, GRMZM2G138750, 100382270* | *putative cytochrome P450 superfamily protein, cytochrome P450 734A1, cytochrome P450 CYP734A8* | *AT2G26710, BAS1/CYP72B1* |
| *Zm00001d039965, GRMZM5G850019, 100282407* | *delta-7-sterol-C5, delta(7)-sterol-C5(6)-desaturase 1* | *AT3G02580, STE1/BUL1/DWF7* |
| **Zm00001d042843, GRMZM2G449033, 100283443** | **steroid reductase DET2, nana plant1, truncated de-etiolated 2** | **AT2G38050, DET2/DWF6** |
| *Zm00001d045563, GRMZM2G093195, 542580, d3* | *cytochrome P450 88A1; ent-kaurenoic acid oxidase, dwarf plant3, siU32579* | *AT1G05160, KAO2;*  *AT2G32440, KAO2* |
| Zm00001d046422, GRMZM2G047684, 100286851, CYP734A7 | cytochrome P450 CYP734A7, cytochrome P450 734A1, putative cytochrome P450 superfamily protein | AT2G26710, BAS1/CYP72B1 |
| *Zm00001d047830, GRMZM2G089803, 103639316* | *cytochrome P450 88A1-like, kaurenoic acid oxidase2, putative cytochrome P450 superfamily protein* | *AT1G05160, KAO2;*  *AT2G32440, KAO2* |
| *Zm00001d050021, GRMZM2G105954, 103653298* | *abscisic acid 8'-hydroxylase 3-like, abscisic acid 8'-hydroxylase3, putative cytochrome P450 superfamily protein* | *AT3G19270, CYP707A4* |
| *Zm00001d051554, GRMZM2G126505, 100274595, umc1299* | *uncharacterized protein LOC100274595, abscisic acid 8'-hydroxylase2, putative cytochrome P450 superfamily protein* | *AT2G29090, CYP707A2; AT4G19230, CYP707A1; AT5G45340, CYP707A3* |
| *Zm00001d051847, GRMZM2G455658, 100276578, natl1, dwfl1, na2-like* | *uncharacterized LOC100276578, 24-methylenecholesterol isomerase/reductase* | *AT3G19820, DWF1/CBB1/DIM* |
| Zm00001d052475, GRMZM2G012391, 100272666, umc1612 | cytochrome P450 90A1, uncharacterized protein LOC100272666, PCO142666, PZA00344, cytochrome P450 CYP90A21, nPZA00344.10, putative cytochrome P450 superfamily protein | AT5G05690, CPD/CBB3/DWF3/CYP90A |
| Zm00001d053617, GRMZM2G107322, 103654817 | cytochrome P450 734A2, cytochrome P450 734A1 | AT2G26710, BAS1 |

Other *A. thaliana* genes known/predicted to be involved in brassinosteroid synthesis according to the TAIR database (https://www.arabidopsis.org/), which do not have orthologs in the maize genome: AT4G36380, ROT3/CYP90C1; AT1G25330, CES; AT1G55940, CYP708A1; AT1G65670, CYP702A1; AT1G73340, ADTO1; AT1G78490, CYP708A3; AT2G42850, CYP718; AT2G45400, BEN1; AT3G30290, CYP702A8; AT4G15300, CYP702A2; AT4G15310, CYP702A3; AT4G15393, CYP702A5; AT4G15396, CYP702A6; AT4G15400, BIA1/ABS-1; AT4G31910, BAT1/PIZ; AT5G36110, CYP716A1; AT5G48000, CYP708A2.

**Maize genes known/predicted to be involved in brassinosteroid signaling.** The gene list was prepared using the NCBI Gene database (https://www.ncbi.nlm.nih.gov/gene/), the Gramene database (http://www.gramene.org/) and the MaizeGDB database (https://www.maizegdb.org/). Genes that were experimentally verified to participate in brassinosteroid signaling in this species (the respective references are given in the main text of the paper) are shown in bold. Additionally, maize orthologs to *Arabidopsis thaliana* genes known to be involved in brassinosteroid signaling according to the TAIR database (https://www.arabidopsis.org/) are also included; these are shown in italics (*Arabidopsis* genes with no known orthologs in the maize genome are listed below the table). Gene orthology was determined using the Putative Orthologous Groups database (http://cas-pogs.uoregon.edu/#/).

| **Gene ID/synonym(s)** | **Gene/protein description** | **Orthologous gene(s) in *A. thaliana*** |
| --- | --- | --- |
| GRMZM2G176604, 100281469 | uncharacterized protein LOC100281469, BRASSINOSTEROID INSENSITIVE 1-associated receptor kinase 1, putative protein kinase superfamily protein | AT2G30940; AT4G01330; AT4G26760 |
| Zm00001d000055, GRMZM2G002515, 103637953 | brassinosteroid LRR receptor kinase BRL2, bri1-like receptor kinase2, putative leucine-rich repeat receptor-like protein kinase family protein, serine/threonine-protein kinase BRI1-like 2 | AT2G01950, BRL2/VH1 |
| Zm00001d000298, GRMZM2G145440, 100281233 | BRASSINOSTEROID INSENSITIVE 1-associated receptor kinase 1 |  |
| *Zm00001d001780, GRMZM2G382104, 100278982* | *probable serine/threonine-protein kinase BSK3, CASP-like protein 1, probable serine/threonine-protein kinase At5g41260-like, putative protein kinase superfamily protein* | *AT1G01740, BSK4; AT1G63500, BSK7; AT4G00710, BSK3; AT5G41260, BSK8* |
| *Zm00001d001982, GRMZM2G388823, 100278153* | *uncharacterized protein LOC100278153, putative HLH DNA-binding domain superfamily protein* | *AT2G43060, IBH1* |
| Zm00001d002121, GRMZM2G072820, 103645865 | transcription factor ILI1, transcription factor PRE3, putative HLH DNA-binding domain superfamily protein | AT1G74500, BS1/PRE3/TMO7; AT5G39860, BRE1/BNQ1 |
| *Zm00001d003256, GRMZM2G093720, 103646298* | *serine/threonine protein phosphatase 2A 57 kDa regulatory subunit B' theta isoform, serine/threonine protein phosphatase 2A 59 kDa regulatory subunit B' eta isoform* | *AT1G13460; AT3G26020* |
| Zm00001d003673, GRMZM2G050861, 100274438, umc1285 | uncharacterized protein LOC100274438, BRASSINOSTEROID INSENSITIVE 1-associated receptor kinase 1, CL12764_1, putative protein kinase superfamily protein | AT4G02630 |
| *Zm00001d004467, GRMZM2G141383, 103646858* | *probable indole-3-pyruvate monooxygenase YUCCA9* | *AT4G28720, YUC8/CKRC2* |
| *Zm00001d004494, GRMZM2G163120* | *TPR repeat-containing thioredoxin TTL1* | *AT2G42580, TTL3/VIT* |
| *Zm00001d005439, GRMZM2G333478, 100281864* | *disulfide oxidoreductase/ monooxygenase/ oxidoreductase* | *AT4G28720, YUC8/CKRC2* |
| *Zm00001d005501, GRMZM2G147885, 103647325* | *serine/threonine protein phosphatase 2A 57 kDa regulatory subunit B' theta isoform, serine/threonine protein phosphatase 2A 59 kDa regulatory subunit B' eta isoform* | *AT1G13460; AT3G26020* |
| *Zm00001d005969, GRMZM2G009478, 100273866* | *putative HLH DNA-binding domain superfamily protein, transcription factor BIM3* | *AT5G08130, BIM1* |
| Zm00001d006677, AC194970.5, 100274111, pco078035 | uncharacterized protein LOC100274111, BES1/BZR family protein BES1/BZR7, pco078035(216) | AT1G19350, BES1/BZR2; AT1G75080, BZR1 |
| Zm00001d007277, GRMZM2G172330, 100285625, cl27483_1 | brassinosteroid LRR receptor kinase, putative leucine-rich repeat transmembrane protein kinase family protein | AT5G48380 |
| Zm00001d007363, GRMZM2G015933, 103648015 | leucine-rich repeat protein 1, BRASSINOSTEROID INSENSITIVE 1-associated receptor kinase 1, somatic embryogenesis receptor kinase 1 |  |
| *Zm00001d007446, GRMZM2G378515, 542285* | *14-3-3-like protein, uncharacterized LOC542285* | *AT5G65430, GRF8; AT5G10450, GRF6* |
| *Zm00001d007968, GRMZM2G378515, 103649501* | *14-3-3-like protein GF14-D, 14-3-3-like protein GF14 nu* | *AT5G10450, GRF6* |
| *Zm00001d007969, GRMZM2G329740, 103648301* | *uncharacterized protein LOC103648301, cold-regulated 413 plasma membrane protein 2* | *AT5G10450, GRF6; AT5G65430, GRF8* |
| *Zm00001d008251, GRMZM2G312419, 100501819* | *uncharacterized protein LOC100501819, putative MYB DNA-binding domain superfamily protein* | *AT5G17800, MYB56/BRAVO* |
| *Zm00001d008617, GRMZM2G085254, 100282789* | *IWS1 C-terminus family protein, IWS1 family protein, transcription elongation factor (TFIIS) family protein* | *AT1G32130, IWS1/HNI9* |
| *Zm00001d008893, GRMZM2G472625, 100193185* | *putative glycogen synthase kinase family protein, shaggy-related protein kinase iota, shaggy-related protein kinase eta* | *AT1G06390, GSK1/BIL2/SK22; AT2G30980, SKdZeta/BIL1; AT4G18710, BIN2/DWF12/SK21/UCU1* |
| *Zm00001d008930, GRMZM2G112659, 100191407* | *uncharacterized protein LOC100191407, TPR repeat-containing thioredoxin TTL1* | *AT2G42580, TTL3/VIT* |
| *Zm00001d009724, GRMZM5G822426, 100193843* | *uncharacterized protein LOC100193843, TPR repeat-containing thioredoxin TTL1* | *AT2G42580, TTL3/VIT* |
| Zm00001d010038, GRMZM2G038780, 100281882 | CASP-like protein 1C2, CASP-like protein 5, UPF0497 membrane protein 6, membrane protein 6, zmCASPL1C2 | AT4G03540, AT1G03700 |
| Zm00001d010465, GRMZM2G139565, 100277664 | uncharacterized protein LOC100277664, putative membrane-associated kinase regulator 1 | AT5G26230, MAKR1 |
| Zm00001d010887, GRMZM2G170798, 100383776 | uncharacterized protein LOC100383776, serine/threonine-protein phosphatase BSL1 | AT4G03080, BSL1 |
| *Zm00001d011352, GRMZM2G128658, 100285724, umc1121* | *uncharacterized protein LOC100285724, electron transporter* | *AT2G42580, TTL3/VIT* |
| **Zm00001d011721, GRMZM2G048294, 103636232** | **brassinosteroid LRR receptor kinase, brassinosteroid insensitive1a, putative leucine-rich repeat receptor-like protein kinase family protein** | **AT4G39400, BRI1/BIN1/CBB2/DWF2** |
| Zm00001d012550, GRMZM2G121820, 100284358 | BRASSINOSTEROID INSENSITIVE 1-associated receptor kinase 1, leucine-rich repeat (LRR) family protein | AT3G43740; AT5G21090 |
| *Zm00001d013078, GRMZM2G099449, 100282793* | *transmembrane BAX inhibitor motif-containing protein 4, BAX inhibitor-1 family protein, transmembrane BAX inhibitor motif protein-containing protein 4* | *AT3G63310, BIL4/LFG2* |
| *Zm00001d013289, GRMZM2G014672, 100284985* | *transmembrane BAX inhibitor motif-containing protein 4, transmembrane BAX inhibitor motif protein-containing protein 4* | *AT3G63310, BIL4/LFG2* |
| Zm00001d013680, GRMZM2G120410, GRMZM2G148539, 100381465 | uncharacterized protein LOC100381465, serine/threonine-protein phosphatase BSL3, putative kelch repeat-containing protein containing ser/thr protein kinase family protein | AT1G08420, BSL2; AT2G27210, BSL3 |
| Zm00001d014705, GRMZM2G089050, 100281505 | nucleic acid binding protein, C3H-type transcription factor, CCCH-type zinc finger protein LIC, putative zinc finger C-x8-C-x5-C-x3-H type family protein | AT1G75340 |
| Zm00001d015581, GRMZM2G118939, 100282372 | BRASSINOSTEROID INSENSITIVE 1-associated receptor kinase 1, protein kinase superfamily protein, putative protein kinase superfamily protein | nemá |
| Zm00001d016197, GRMZM2G146794, 100285968 | BRASSINOSTEROID INSENSITIVE 1-associated receptor kinase 1, putative LRR receptor-like serine/threonine-protein kinase, putative leucine-rich repeat receptor-like protein kinase family protein | AT5G45780 |
| Zm00001d016231, GRMZM2G369018, 103627031 | protein BZR1 homolog 4, BES1/BZR family protein BES1/BZR8, BZR transcription factor, protein BRASSINAZOLE-RESISTANT 1 | AT1G78700, BEH4; AT4G18890, BEH3 |
| *Zm00001d016660, GRMZM2G326111, 103627222* | *peptidyl-prolyl cis-trans isomerase-like, peptidyl-prolyl isomerase1, putative peptidyl-prolyl cis-trans isomerase family protein* | *AT4G38740, ROC1* |
| *Zm00001d016928, GRMZM2G115420, 542016, serk2* | *somatic embryogenesis receptor-like kinase 2, CL444_1(430), CL444_1b, QAH2a06, somatic embryogenesis receptor kinase 2, gnp_QAH2a06a, gpm342a, putative leucine-rich repeat receptor-like protein kinase family protein, umc2304* | *AT1G34210, SERK2; AT1G71830, SERK1* |
| Zm00001d017612, GRMZM2G071277, 100284526 | RING zinc finger protein-like, brassinosteroid-responsive RING-H2, putative RING zinc finger domain superfamily protein |  |
| *Zm00001d017908, GRMZM2G317450, 100279738* | *transcription factor BIM3, uncharacterized protein LOC100279738, putative HLH DNA-binding domain superfamily protein* | *AT5G08130, BIM1* |
| Zm00001d018344, GRMZM2G027439, 100284386 | BRASSINOSTEROID INSENSITIVE 1-associated receptor kinase 1, receptor like protein 57 | AT3G49750 |
| *Zm00001d018369, GRMZM5G879749, 100284338, cl8680_1* | *uncharacterized protein LOC100284338, serine carboxypeptidase 24, cl8680_1(454), serine carboxypeptidase K10B2.2* | *AT2G24000, BRS1/SCPL24; AT3G02110, SCPL25; AT4G30610, BRS1/SCPL24* |
| *Zm00001d018856, GRMZM2G097856, 103633695* | *14-3-3-like protein GF14-D* | *AT5G10450, GRF6; AT5G65430, GRF8* |
| *Zm00001d018938, GRMZM2G132568, 100191683, pco086679* | *uncharacterized protein LOC100191683, PZA01613, nPZA01613-1, pco086679(535), transmembrane BAX inhibitor motif protein-containing protein 4* | *AT3G63310, BIL4/LFG2* |
| *Zm00001d019363, GRMZM2G106141, 100382877* | *uncharacterized protein LOC100382877, serine/threonine protein phosphatase 2A 59 kDa regulatory subunit B' eta isoform* | *AT1G13460; AT3G26020* |
| *Zm00001d019527, GRMZM2G132489, 100281459, umc2526* | *uncharacterized protein LOC100281459, disulfide oxidoreductase/monooxygenase/ oxidoreductase, Yucca5* | *AT4G28720, YUC8/CKRC2* |
| Zm00001d019950, GRMZM2G015011, 103632502 | DNA topoisomerase 6 subunit B | AT3G20780, TOP6B/BIN3/HLQ/RHL3 |
| Zm00001d019983, GRMZM2G092604, 103632518 | brassinosteroid LRR receptor kinase BRL1, bri1-like receptor kinase1, putative leucine-rich repeat receptor-like protein kinase family protein, receptor-like protein kinase BRI1-like 3 | AT1G55610, BRL1 |
| Zm00001d020256, GRMZM2G085604, 100382146 | uncharacterized protein LOC100382146, P-loop containing nucleoside triphosphate hydrolase superfamily protein | AT4G10620 |
| Zm00001d020680, GRMZM5G890224, 103643406 | PHD finger protein MALE STERILITY 1-like, PHD finger protein MALE STERILITY 1, male sterile 7 | AT5G22260, MS1 |
| *Zm00001d020725, GRMZM2G135400, 103632857* | *probable BRI1 kinase inhibitor 1, BRI1 kinase inhibitor 1* | *AT5G42750, BKI1* |
| *Zm00001d020834, GRMZM2G089501, 100191909* | *putative HLH DNA-binding domain superfamily protein, transcription factor BIM2* | *AT5G08130, BIM1* |
| *Zm00001d021350, GRMZM2G171884, 100381482* | *uncharacterized protein LOC100381482, chaperone DnaJ-domain superfamily protein* | *AT2G42080* |
| Zm00001d021839, GRMZM2G081949, 100284456, pco127157 | uncharacterized protein LOC100284456, remorin family protein , DNA binding protein, pco127157(577) | AT3G57540, AT2G41870 |
| *Zm00001d021885, GRMZM2G305211* | *14-3-3-like protein* | *AT5G10450, GRF6; AT5G65430, GRF8* |
| Zm00001d021927, GRMZM2G102514, 103633350 | BES1/BZR1 protein, BES1/BZR family protein BES1/BZR10, protein BZR1 homolog 1-like | AT1G19350, BES1/BZR2; AT1G75080, BZR1/BES1; AT3G50750, BEH1; AT4G36780, BEH2 |
| *Zm00001d022239, GRMZM2G159034, 100382164, pco131426* | *uncharacterized protein LOC100382164, VH1-interacting kinase, putative protein kinase superfamily protein* | *AT1G14000, VIK* |
| *Zm00001d024166, GRMZM2G145909, 100278375* | *uncharacterized protein LOC100278375, putative HLH DNA-binding domain superfamily protein* | *AT4G30410, IBL1* |
| *Zm00001d024430, GRMZM2G384439, 103641205* | *somatic embryogenesis receptor kinase 2-like, putative leucine-rich repeat receptor-like protein kinase family protein* | *AT1G34210, SERK2; AT1G71830, SERK1* |
| *Zm00001d025005, GRMZM2G159393, 100281570* | *disulfide oxidoreductase/ monooxygenase/ oxidoreductase* | *AT4G28720, YUC8/CKRC2* |
| *Zm00001d025580, GRMZM5G870959, 542015, serk1* | *somatic embryogenesis receptor-like kinase 1, PZA03709, PZA03710, PZA03711, PZA03713, ZmSERK1, nPZA03713.1, putative leucine-rich repeat receptor-like protein kinase family protein* | *AT1G34210, SERK2; AT1G71830, SERK1* |
| *Zm00001d025714, GRMZM2G058525, 100501635* | *uncharacterized protein LOC100501635, serine/threonine protein phosphatase 2A 59 kDa regulatory subunit B' eta isoform* | *AT1G13460; AT3G26020* |
| *Zm00001d026510, GRMZM2G084576, 100275788, umc2507* | *uncharacterized protein LOC100275788, putative HLH DNA-binding domain superfamily protein* | *AT2G43060, IBH1* |
| Zm00001d026653, 100282637 | protein kinase APK1B, BR-signaling kinase 3 | AT1G01740, BSK4; AT1G63500, BSK7; AT4G00710, BSK3; AT5G41260, BSK8 |
| *Zm00001d027523, GRMZM2G099598, 100273485* | *putative protein kinase superfamily protein, putative serine/threonine-protein kinase* | *AT1G50990, BSK11; AT2G17090, SPP/BSK12; AT4G35230, BSK1* |
| Zm00001d027587, GRMZM2G152172, 100381940 | uncharacterized LOC100381940, BES1/BZR family protein BES1/BZR9 | AT1G78700, BEH4; AT4G18890, BEH3 |
| *Zm00001d027855, GRMZM2G035156, 103631776* | *transcription factor ILI6, transcription factor PRE3, putative HLH DNA-binding domain superfamily protein* | *AT1G74500, BS1/PRE3/TMO7; AT5G39860, BRE1/BNQ1* |
| Zm00001d027886, GRMZM2G064732, 100284718, IDP407 | uncharacterized protein LOC100284718, compact plant2, PCO117747, guanine nucleotide-binding protein alpha-1 subunit | AT2G26300, GPALPHA1/GPA1 |
| Zm00001d027957, GRMZM2G059102, 542034, TIDP3516 | MADS-box transcription factor 47, MADS-transcription factor 68, putative MADS-box transcription factor family protein | AT2G22540; AT4G24540 |
| *Zm00001d028701, AC217300.3, 103634484* | *probable serine/threonine-protein kinase PBL7, putative protein kinase superfamily protein, putative serine/threonine-protein kinase RLCKVII* | *AT5G02800, CDL1* |
| Zm00001d029597, GRMZM2G084587, 100282539 | BRASSINOSTEROID INSENSITIVE 1-associated receptor kinase 1, leucine-rich repeat (LRR) family protein |  |
| *Zm00001d029785, GRMZM2G026767, 100280241* | *putative protein kinase superfamily protein, putative serine/threonine-protein kinase* | *AT1G50990, BSK11; AT2G17090, SPP/BSK12; AT4G35230, BSK1* |
| Zm00001d030021, GRMZM2G169080, 103637689 | probable serine/threonine-protein kinase At4g35230, BR-signaling kinase 2, putative protein kinase superfamily protein | AT5G46570, BSK2 |
| Zm00001d031088, GRMZM2G028700, 100192777 | putative kelch repeat-containing protein containing ser/thr protein kinase family protein, serine/threonine-protein phosphatase | AT1G08420, BSL2; AT2G27210, BSL3 |
| *Zm00001d031798, GRMZM2G334639, 103643150* | *probable BRI1 kinase inhibitor 1, BRI1 kinase inhibitor 1* | *AT5G42750, BKI1* |
| Zm00001d033217, GRMZM2G070323, 103643688 | serine/threonine-protein phosphatase BSL2 homolog, serine/threonine-protein phosphatase BSL3 | AT1G08420, BSL2; AT2G27210, BSL3 |
| Zm00001d033228, GRMZM2G080828, 100191445 | putative ornithine aminotransferase protein, ornithine aminotransferase mitochondrial | AT5G46180 |
| *Zm00001d033392, GRMZM2G357081* | *shaggy-related protein kinase iota* | *AT1G06390, GSK1/BIL2/SK22; AT2G30980, SKdZeta/BIL1; AT4G18710, BIN2/DWF12/SK21/UCU1* |
| *Zm00001d033990, GRMZM2G128617, 103643997* | *protein LIFEGUARD 2, BI1-like protein, Bax inhibitor-1 family protein* | *AT3G63310, BIL4/LFG2* |
| *Zm00001d034432, GRMZM2G074404, 100283434* | *transmembrane BAX inhibitor motif-containing protein 4, Bax inhibitor-1 family protein, transmembrane BAX inhibitor motif protein-containing protein 4* | *AT3G63310, BIL4/LFG2* |
| *Zm00001d034623, GRMZM2G076114, 103645709* | *glycogen synthase kinase-3 homolog MsK-3, putative glycogen synthase kinase family protein* | *AT1G06390, GSK1/BIL2/SK22; AT2G30980, SKdZeta/BIL1; AT4G18710, BIN2/DWF12/SK21/UCU1* |
| *Zm00001d034817, GRMZM2G369912, 732821, PPP2R5B* | *protein phosphatase 2A regulatory subunit B', serine/threonine protein phosphatase 2A 59 kDa regulatory subunit B' eta isoform, protein phosphatase 2A regulatory subunit B* | *AT1G13460; AT3G26020* |
| *Zm00001d035140, GRMZM2G009661, 103628994* | *serine/threonine protein phosphatase 2A 57 kDa regulatory subunit B' theta isoform, serine/threonine protein phosphatase 2A 59 kDa regulatory subunit B' eta isoform* | *AT1G13460; AT3G26020* |
| Zm00001d037110, GRMZM2G120371, 100280504 | G-protein coupled receptor, G-protein coupled receptor 1 | AT1G48270, GCR1 |
| Zm00001d037297, GRMZM2G349665, 100281584 | BRASSINOSTEROID INSENSITIVE 1-associated receptor kinase, putative leucine-rich repeat receptor-like protein kinase family protein |  |
| Zm00001d037413, GRMZM2G101874, 100285406 | microtubule-associated protein MAP65-1a, 65-kDa microtubule-associated protein 1 | AT4G26760 |
| *Zm00001d037950, GRMZM2G049342, 542211, cl3114_1(600)* | *uncharacterized protein LOC542211, serine/threonine-protein kinase TOR, target of rapamycin* | *AT1G50030, TOR* |
| Zm00001d038195, GRMZM2G121565, 100281404 | BRASSINOSTEROID INSENSITIVE 1-associated receptor kinase 1 , putative inactive leucine-rich repeat receptor-like protein kinase, putative leucine-rich repeat transmembrane protein kinase family protein |  |
| *Zm00001d039042, GRMZM2G366778, 103631640* | *14-3-3-like protein GF14-D, 14-3-3-like protein GF14 nu* | *AT5G10450, GRF6; AT5G65430, GRF8* |
| *Zm00001d039362, GRMZM2G135089, GRMZM2G166612, 103653343* | *TPR repeat-containing thioredoxin TTL1* | *AT2G42580, TTL3/VIT* |
| *Zm00001d039407, GRMZM2G045330, 100282757* | *shaggy-related protein kinase eta, Shaggy-related protein kinase iota, putative glycogen synthase kinase family protein* | *AT1G06390, GSK1/BIL2/SK22; AT2G30980, SKdZeta/BIL1; AT4G18710, BIN2/DWF12/SK21/UCU1* |
| Zm00001d039439, GRMZM5G852801, GRMZM6G287292, 100194109 | protein BRASSINAZOLE-RESISTANT 1, BES1/BZR family protein BES1/BZR11, BES1/BZR family protein BES1/BZR2, brassinazole-resistant 1 protein |  |
| Zm00001d039635, GRMZM5G868061 | BZR-transcription factor 6, BES1/BZR1 homolog protein 4 |  |
| *Zm00001d039871, GRMZM2G100881, 103649783* | *protein IWS1 homolog 1, transcription elongation factor (TFIIS) family protein* | *AT1G32130, IWS1/HNI9* |
| *Zm00001d040423, GRMZM2G111731, 100381481* | *MYB-transcription factor, myb domain protein 56, putative MYB DNA-binding domain superfamily protein* | *AT5G17800, MYB56/BRAVO* |
| *Zm00001d040426, GRMZM2G083239, 103650011* | *transcription factor CSA, myb domain protein 56* | *AT5G17800, MYB56/BRAVO* |
| Zm00001d041307, GRMZM2G009593, 103650267 | serine/threonine-protein phosphatase BSL2 homolog, serine/threonine-protein phosphatase BSL3 | AT1G08420, BSL2; AT2G27210, BSL3 |
| Zm00001d043149, GRMZM5G815009, 100282767 | BRASSINOSTEROID INSENSITIVE 1-associated receptor kinase 1, leucine-rich repeat (LRR) family protein | AT3G43740; AT5G21090 |
| Zm00001d043634, GRMZM6G437417, 103642983 | brassinosteroid LRR receptor kinase BRI1, BRI-like receptor, protein BRASSINOSTEROID INSENSITIVE 1, brassinosteroid receptor | AT4G39400, BRI1/BIN1/CBB2/DWF2 |
| *Zm00001d044142, GRMZM2G364528, 100275101* | *uncharacterized protein LOC100275101* | *AT2G42870, PAR1/HLH1; AT3G58850, PAR2/HLH2* |
| *Zm00001d044479, GRMZM2G093436, 100502390* | *uncharacterized protein LOC100502390, TPR repeat-containing thioredoxin TTL1* | *AT2G42580, TTL3/VIT* |
| Zm00001d044839, GRMZM5G878346, 100281850, umc2393 | uncharacterized protein LOC100281850, transcription factor PRE3, DNA binding protein |  |
| Zm00001d045310, GRMZM2G020216, 541618, mpk2 | MAP kinase 2, MAP kinase 5, MAP kinase2, MPK6 - putative MAPK, PCO143674, PCO143674(662), PCO143674b, putative MAP kinase family protein | AT2G43790; AT4G26760 |
| Zm00001d045507, AC234164.1, 100283946 | ATP synthase D chain, mitochondrial, scarecrow-like protein 28 | AT1G63100 |
| Zm00001d045568, GRMZM2G367650, 100284868 | RING zinc finger protein-like, brassinosteroid-responsive RING-H2, putative RING zinc finger domain superfamily protein | AT1G63840; AT3G61460, BRH1; AT5G41400 |
| Zm00001d045728, GRMZM2G145720, 100382590 | putative leucine-rich repeat receptor-like protein kinase family protein, Protein NSP-INTERACTING KINASE 1, brassinosteroid insensitive1-associated receptor kinase like1 |  |
| Zm00001d046305, GRMZM5G812774, 100216929 | BES transcription factor, BES1/BZR family protein BES1/BZR1, MYBGA transcription factor, protein BRASSINAZOLE-RESISTANT 1, brassinazole-resistant 1 protein | AT1G78700, BEH4; AT4G18890, BEH3 |
| *Zm00001d046867, GRMZM2G435001, 103638849* | *transcription factor IBH1-like 1, putative HLH DNA-binding domain superfamily protein* | *AT4G30410, IBL1* |
| Zm00001d047053, GRMZM2G054634, 100282511 | ATP binding protein, BR-signaling kinase 2, putative protein kinase superfamily protein | AT5G46570, BSK2 |
| Zm00001d047217, GRMZM6G548844, 100273769, pco061649(751) | uncharacterized protein LOC100273769, 5-hydroxyisourate hydrolase, carrier/ steroid binding protein | AT5G58220, TTL/ALNS |
| Zm00001d047842, GRMZM2G479243, 100281527, IDP1669 | uncharacterized protein LOC100281527, BRASSINOSTEROID INSENSITIVE 1-associated receptor kinase 1, putative leucine-rich repeat receptor-like protein kinase family protein | AT2G35620 |
| *Zm00001d048345, GRMZM2G127050, 100192644* | *uncharacterized protein LOC100192644, ATP binding protein, putative protein kinase superfamily protein* | *AT1G50990, BSK11; AT2G17090, SPP/BSK12; AT4G35230, BSK1* |
| Zm00001d048404, AC149475.2, 100381553 | uncharacterized protein LOC100381553, AC149475.2_FG005, putative lysine-specific demethylase ELF6 | AT5G04240, ELF6 |
| *Zm00001d048868, GRMZM2G145213, 100193401* | *14-3-3-like protein, 14-3-3-like protein GF14 epsilon* | *AT1G22300, GRF10* |
| Zm00001d048877, GRMZM5G867798, 100280511 | BRASSINOSTEROID INSENSITIVE 1-associated receptor kinase 1, putative LRR receptor-like serine/threonine-protein kinase, putative leucine-rich repeat receptor-like protein kinase family protein |  |
| *Zm00001d049642, GRMZM2G049127, 100279734* | *uncharacterized protein LOC100279734, serine/threonine protein phosphatase 2A 59 kDa regulatory subunit B' eta isoform* | *AT1G13460; AT3G26020* |
| Zm00001d050132, GRMZM2G438007, 103653351 | bifunctional aspartokinase/homoserine dehydrogenase 1, chloroplastic, bri1-like receptor kinase3 | AT1G55610, BRL1 |
| *Zm00001d050635, GRMZM2G162388, 103653580* | *peptidyl-prolyl cis-trans isomerase-like, peptidyl-prolyl cis-trans isomerase, putative peptidyl-prolyl cis-trans isomerase family protein* | *AT4G38740, ROC1* |
| *Zm00001d050868, GRMZM2G150024, 542670* | *somatic embryogenesis receptor-like kinase 3, CL444_1, CL444_1a, QAH2a06, ZmSERK3, putative leucine-rich repeat receptor-like protein kinase family protein, somatic embryogenesis receptor-like kinase3* | *AT1G34210, SERK2; AT1G71830, SERK1* |
| *Zm00001d052054, GRMZM2G155499, 100274484* | *putative HLH DNA-binding domain superfamily protein, sequence-specific DNA binding transcription factor* | *AT4G30410, IBL1* |
| Zm00001d052323, GRMZM2G089819, 100281413 | brassinosteroid LRR receptor kinase, meristematic receptor-like kinase, putative leucine-rich repeat receptor-like protein kinase family protein | AT3G51740; AT3G56100 |
| *Zm00001d052423, GRMZM2G035042, 100281405* | *IMP dehydrogenase/GMP reductase, GTP-binding protein BRASSINAZOLE INSENSITIVE PALE GREEN 2 chloroplastic* | *AT3G57180, BPG2* |
| Zm00001d052944, GRMZM2G062772, 732824, CCamK | putative calcium/calmodulin dependent protein kinase, calcium/calmodulin dependent protein kinase1, putative calcium-dependent protein kinase family protein |  |
| *Zm00001d053090, GRMZM2G106424, 100273134* | *uncharacterized protein LOC100273134, 14-3-3-like protein* | *AT5G10450, GRF6; AT5G65430, GRF8* |
| Zm00001d053543, GRMZM2G307241, 103654774 | protein BZR1 homolog 3, BES1/BZR family protein BES1/BZR3, protein BRASSINAZOLE-RESISTANT 1 | AT1G78700, BEH4; AT4G18890, BEH3 |
| Zm00001d053617, GRMZM2G107322, 103654817 | cytochrome P450 734A2, cytochrome P450 734A1 | AT2G26710, BAS1 |
| Zm00001d053725, GRMZM2G125308, 100216921 | putative protein kinase superfamily protein, BRASSINOSTEROID INSENSITIVE 1-associated receptor kinase 1, protein kinase superfamily protein |  |

Other *A. thaliana* genes known/predicted to be involved in brassinosteroid signaling according to the TAIR database (https://www.arabidopsis.org/), which do not have orthologs in the maize genome: AT1G03445, BSU1; AT1G35160, GF14 PHI; AT1G67160; AT1G78300, GRF2; AT2G13790, SERK4/BAK7/BKK1; AT2G18300, HBI1; AT3G03730; AT3G09240, BSK9; AT3G09880, ATB BETA; AT3G21650; AT3G26940, CDG1; AT3G46290, HERK1; AT3G51550, FER; AT3G54030, BSK6; AT4G12810, KIB1; AT4G12820; AT4G15415, ATB GAMMA; AT4G33430, BAK1/ELG/RKS10/SERK3; AT5G01060, BSK10; AT5G03470, ATB ALPHA; AT5G54380, THE1; AT5G59010, BSK5.

**Main experimental aspects of the transcriptome studies performed with drought-stressed maize, which showed differential expression of genes involved in brassinosteroid biosynthesis, catabolism/homeostasis or signaling** (the lists of these genes are given in Tables S7 and S8). For further details of these studies see the respective references (their list is at the end of this file). DAS … days after sowing, PEG … polyethylene glycol, RH … relative air humidity, RNAseq … RNA sequencing, RWC … relative water content.

| **Cultivar(s)** | **Analysed organ** | **Plant age/developmental stage at the beginning of stress → at the time of the sampling for the transcriptome analysis** | **Cultivation conditions** | **Drought simulation** | **Transcriptome analysis** | **Data depository** | **Reference** |
| --- | --- | --- | --- | --- | --- | --- | --- |
| Han21, Ye478 inbred lines | Shoot | 14 DAS (V3) → moderate or severe drought (*see the column Drought simulation*) | Growth chamber (30/26°C, 16/8 h photoperiod, 300 μmol m^-2^ s^-1^, 30–50% RH, containers with sand) | Withholding water; RWC 66-72% (moderate drought) or 41-53% (severe drought) | GeneChip Maize Genome Arrays (Affymetrix) | NCBI GEO, GSE16567 | Zheng *et al.* 2010 |
| B73 inbred line | Primary roots | 4-5 DAS → 6 h or 24 h after the start of drought simulation | Growth chamber (28/21°C, 16/8 h photoperiod, paper rolls soaked with water) | PEG8000 treatment, -0.2 MPa (mild drought) or -0.8 MPa (severe drought); plant water status unknown | RNAseq | NCBI SRA, SRP032921 | Opitz *et al.* 2014 |
| B73 inbred line | Leaf (not stated which one) | 14 DAS → 2 h after the start of drought simulation | Greenhouse (conditions not stated, pots with soil) | Removal from soil, exposure to air drying for 2 h at 22°C; RWC approx. 78% | RNAseq | NCBI GEO, GSE48507 | Ding *et al.* 2014 |
| ZD619 inbred line | Leaf (mix of the top 3 leaves), stem, roots | 21 DAS (V2/V3 stage) → approx. 10 d after the start of drought simulation | Greenhouse (25/20°C, 16/8 h photoperiod, pots with soil: vermiculite: nutrient soil mix 1:1:1) | Withholding water until soil RWC reached 35%; plant water status unknown | RNAseq | NCBI GEO, GSE71377 | Liu *et al.* 2015 |
| B73 inbred line (wt), *rpd1-1/rmr6* mutant | Leaf (the youngest one) | V5/V6 stage → 51 d after the start of drought simulation | Greenhouse (28-30/20-22°C, 60-80% RH, pots with soil) | Watering with 25% of disposable water for 10 d, then 7 d with 75%, 3 times repeated; plant water status unknown | RNAseq | NCBI GEO, GSE71046 | Forestan *et al.* 2016 |
| B73 inbred line | Leaf (the youngest fully developed one), ear, tassel | V8 stage → V12, V14, V18, R1 stages (*i.e.*, 11, 18, 27, 32 d after the start of drought simulation) | Field (Woodland, California, USA) | Withholding water; plant water status unknown | RNAseq | NCBI GEO, GSE71723 | Thatcher *et al.* 2016^1^ |
| LV28, H082183 inbred lines | Leaf (not stated which one) | 21 DAS (V4 stage) → 27 d (moderate drought) or 46 d (severe drought) | Field (Urumqi, Xinjiang, China) | Withholding water; leaf RWC 84-90% (moderate drought) or 82-84% (severe drought) | RNAseq | NCBI SRA, SRP102142 | Zhang *et al.* 2017 |

**Differential expression of genes known/predicted to be involved in brassinosteroid biosynthesis and/or catabolism/homeostasis revealed by the transcriptome studies performed in drought-stressed maize**. Only the genes with log2 fold change ratio (drought/control) ≥ 1 or ≤ -1 and false discovery rate (FDR)-adjusted p value ≤ 0.05 were considered as statistically differentially expressed and are included in this table. The description of genes and the meaning of italics and bold in the column Gene ID is given in the legend to Table S7. Individual cells show the log2 fold change ratios inferred from the data supplied by the studies listed in Table S9. Red colour means that the expression of the respective gene increased, blue colour means that its expression was reduced in drought-stressed plants compared to the control ones (light and dark tones differentiate between log2 fold change ratios ≥ 1 or ≤ -1 and ≥ 2 or ≤ -2. In most cases several experimental variants (different cultivars, analysed organs, drought intentisies or sampling times) were examined, the description of these variants can be found in Table S9. MD … moderate or mild drought, SD … severe drought.

| **Gene ID** | **Zheng *et al.* 2010** | | | | **Opitz *et al.* 2014** | | | | **Ding *et al.* 2014** | **Liu *et al.* 2015** | | | **Forestan *et al.* 2016** | | **Thatcher *et al.* 2016** | | | | | | | | | | | | **Zhang *et al.* 2017** | | | | |
| --- | --- | --- | --- | --- | --- | --- | --- | --- | --- | --- | --- | --- | --- | --- | --- | --- | --- | --- | --- | --- | --- | --- | --- | --- | --- | --- | --- | --- | --- | --- | --- |
|  | **MD** | | **SD** | | **MD** | | **SD** | |  | **Leaf** | **Stem** | **Roots** | **wt** | **Mutant** | **Leaf** | | | | **Ear** | | | | **Tassel** | | | | **MD** | | | **SD** | |
|  | **Han21** | **Ye478** | **Han21** | **Ye478** | **24 h** | **6 h** | **24 h** | **6 h** |  |  |  |  |  |  | **V12** | **V14** | **V18** | **R1** | **V12** | **V14** | **V18** | **R1** | **V12** | **V14** | **V18** | **R1** | **LV28** | **H082183** | **LV28** | | **H082183** |
| *Zm00001d002629* |  |  |  |  |  |  | -1.3 |  |  |  |  |  |  |  |  |  |  |  |  |  |  |  |  |  |  |  |  |  |  | |  |
| Zm00001d003349 | -1.1 | -1.2 | -1.5 | -1.4 |  |  |  |  |  |  |  |  |  |  |  |  |  |  | -1.4 |  |  | 1.1 |  |  |  | 1.1 |  |  |  | |  |
| Zm00001d004957 |  |  |  |  |  |  |  |  | -2.3 |  |  |  |  |  |  |  |  |  |  |  |  | -2.2 |  |  |  |  |  |  |  | |  |
| *Zm00001d005889* |  |  |  |  | 1.5 |  | 3.0 |  |  | -3.3 |  | -2.8 | 2.1 |  | -1.0 |  | -1.1 |  |  |  |  | 2.6 |  |  |  |  |  |  |  | |  |
| *Zm00001d008569* |  |  | -1.3 | -1.5 |  |  |  |  |  |  |  |  |  |  |  |  |  |  |  |  |  |  |  |  |  |  |  |  |  | |  |
| *Zm00001d011117* |  |  |  |  | -1.3 |  | -1.9 | -1.1 |  |  | -2.6 |  |  |  |  |  |  |  |  |  |  | -1.2 |  |  |  |  |  |  |  | |  |
| *Zm00001d013629* |  |  |  |  |  |  | -1.0 |  |  |  | 2.7 |  |  |  | -3.0 | -2.3 | -3.8 | -6.6 |  |  |  |  |  |  |  |  |  |  |  | |  |
| *Zm00001d013720* |  |  |  |  |  |  | -1.9 |  |  |  | -2.2 |  |  |  |  |  |  |  |  |  |  |  |  |  |  |  |  |  |  | |  |
| **Zm00001d014887** |  |  | -1.1 |  |  |  |  |  |  |  |  |  |  |  |  |  |  |  |  |  |  |  |  |  |  |  |  |  |  | |  |
| *Zm00001d017582* |  |  |  |  |  |  | -1.4 |  |  |  |  |  |  |  |  |  |  |  |  |  |  |  |  |  |  |  |  |  |  | |  |
| *Zm00001d017762* |  |  |  |  |  |  |  |  | 3.3 | 4.0 |  |  |  |  |  |  |  |  |  |  |  | -2.3 | -1.1 | -1.7 |  |  |  |  |  | |  |
| *Zm00001d020717* | 1.5 |  | 1.7 |  |  |  |  |  | 3.1 |  |  |  | 1.6 |  |  |  |  |  | 1.1 |  |  |  |  |  |  |  |  |  |  | |  |
| **Zm00001d028325** |  |  |  |  |  |  |  |  |  |  |  |  | 1.2 |  |  |  |  |  |  |  |  |  |  |  |  |  | 1.2 | -1.2 |  | |  |
| **Zm00001d033180** |  |  |  |  |  |  |  |  |  |  | -2.4 |  |  |  |  |  |  |  | -1.5 |  |  | -1.2 | -1.1 |  |  |  |  |  |  | |  |
| Zm00001d037745 |  |  |  |  |  |  |  |  |  |  | 1.7 |  | 2.0 |  |  |  |  |  |  |  |  |  |  |  |  |  |  |  |  | |  |
| Zm00001d039453 |  |  |  |  |  |  |  |  |  |  |  |  |  |  |  |  |  |  |  |  |  | -1.9 |  |  |  |  |  |  |  | |  |
| *Zm00001d039650* |  |  |  |  |  |  |  |  |  |  |  |  |  |  |  |  |  |  |  | -1.3 |  | -1.3 |  |  |  |  |  |  |  | |  |
| *Zm00001d039965* |  |  |  |  |  |  | 1.4 |  |  |  |  |  |  |  |  |  |  |  |  |  |  |  |  |  |  |  |  |  |  | |  |
| *Zm00001d045563* |  |  |  |  |  |  |  |  |  |  |  |  |  |  |  |  |  |  |  |  | 1.2 | 1.4 |  |  | 1.5 | -1.1 |  |  |  | |  |
| *Zm00001d047830* |  |  |  |  | -1.8 |  | -2.0 | -1.0 |  |  |  | 2.9 |  |  |  |  |  |  |  |  |  |  |  |  |  |  |  |  |  | |  |
| *Zm00001d050021* |  |  |  |  | 1.0 |  |  | 1.2 |  | -3.1 |  |  |  |  |  |  | 4.6 | 3.0 |  | -1.2 |  | 1.6 | 1.7 |  |  |  |  |  |  | |  |
| *Zm00001d051554* | 1.0 | 1.2 | 2.2 | 2.9 | 1.2 |  | 2.7 |  | 1.6 |  |  |  |  |  |  |  |  | -1.3 |  |  | -1.3 |  |  |  |  |  |  |  |  | |  |

**Differential expression of genes known/predicted to be involved in brassinosteroid signaling revealed by the transcriptome studies performed in drought-stressed maize**. Only the genes with log2 fold change ratio (drought/control) ≥ 1 or ≤ -1 and false discovery rate (FDR)-adjusted p value ≤ 0.05 were considered as statistically differentially expressed and are included in this table. The description of genes and the meaning of italics and bold in the column Gene ID is given in Table S8. Individual cells show the log2 fold change ratios inferred from the data supplied by the studies listed in Table S9. Red colour means that the expression of the respective gene increased, blue colour means that its expression was reduced in drought-stressed plants compared to the control ones (light and dark tones differentiate between log2 fold change ratios ≥ 1 or ≤ -1 and ≥ 2 or ≤ -2. In most cases several experimental variants (different cultivars, analysed organs, drought intentisies or sampling times) were examined, the description of these variants can be found in Table S9. MD … moderate or mild drought, SD … severe drought.

| **Gene ID** | **Zheng *et al.* 2010** | | | | **Opitz *et al.* 2014** | | | | **Ding *et al.* 2014** | **Liu *et al.* 2015** | | | **Forestan *et al.* 2016** | | **Thatcher *et al.* 2016** | | | | | | | | | | | | **Zhang *et al.* 2017** | | | | |
| --- | --- | --- | --- | --- | --- | --- | --- | --- | --- | --- | --- | --- | --- | --- | --- | --- | --- | --- | --- | --- | --- | --- | --- | --- | --- | --- | --- | --- | --- | --- | --- |
|  | **MD** | | **SD** | | **MD** | | **SD** | |  | **Leaf** | **Stem** | **Roots** | **WT** | **Mutant** | **Leaf** | | | | **Ear** | | | | **Tassel** | | | | **MD** | | | **SD** | |
|  | **Han21** | **Ye478** | **Han21** | **Ye478** | **24 h** | **6 h** | **24 h** | **6 h** |  |  |  |  |  |  | **V12** | **V14** | **V18** | **R1** | **V12** | **V14** | **V18** | **R1** | **V12** | **V14** | **V18** | **R1** | **LV28** | **H082183** | **LV28** | | **H082183** |
| Zm00001d000298 |  |  |  |  |  |  |  |  |  |  |  |  |  |  |  |  | -1.7 | -4 |  |  |  |  |  |  |  |  |  |  |  | |  |
| *Zm00001d001982* |  |  |  |  |  |  |  |  |  |  |  |  |  |  |  |  |  |  | -1.8 |  |  |  |  |  |  |  | -1.3 |  |  | |  |
| Zm00001d002121 | -1.3 | -2.3 | -3.4 | -2.5 |  |  |  |  |  |  |  |  |  | 1.5 |  |  |  |  |  |  |  |  |  |  |  |  |  |  |  | |  |
| *Zm00001d003256* |  |  |  |  |  |  |  |  |  |  |  |  |  |  |  |  |  |  |  |  |  |  | -1.0 |  |  |  |  |  |  | |  |
| Zm00001d003673 |  |  | -1.3 | -1.3 |  |  |  |  |  | -1.6 |  |  |  |  |  | 1.1 |  | 1.0 | 1.7 |  |  |  |  |  |  |  |  |  |  | |  |
| *Zm00001d004467* |  |  |  |  |  |  |  |  |  |  | -2.6 | 4.4 |  |  |  |  |  |  |  |  |  |  |  |  | 1.1 |  |  |  |  | |  |
| *Zm00001d005439* |  |  |  |  |  |  |  |  |  |  |  | -1.8 | 3.7 |  |  |  |  |  | -2.0 |  |  |  |  |  |  |  |  |  |  | |  |
| *Zm00001d005969* |  |  |  |  |  |  |  |  | 1.3 |  |  |  |  |  |  |  |  |  | -1.0 |  |  |  |  |  |  |  |  |  |  | |  |
| Zm00001d006677 |  |  |  |  |  |  |  |  | 1.2 | -1.0 |  |  | 2.0 |  |  |  |  |  |  | 1.0 |  |  |  |  |  |  |  |  |  | |  |
| *Zm00001d007446* | 1.1 | 1.6 | 1.3 | 2.0 |  |  |  |  | 3.8 |  |  |  |  |  |  |  |  |  |  |  |  |  |  |  |  |  |  |  |  | |  |
| *Zm00001d008251* |  |  |  |  |  |  |  |  |  |  |  |  | 3.1 |  |  |  |  |  |  |  |  |  |  |  |  |  |  |  |  | |  |
| *Zm00001d008617* |  |  |  | 1.1 |  |  |  |  |  |  |  |  |  |  |  |  |  |  |  |  |  |  |  |  |  |  |  |  |  | |  |
| *Zm00001d008930* |  |  |  |  |  |  |  |  |  |  |  |  |  |  |  |  |  |  |  |  |  |  | -1.4 |  |  |  |  |  |  | |  |
| *Zm00001d009724* |  |  | -1.5 |  |  |  |  |  |  |  |  |  |  |  |  |  |  |  |  |  |  |  |  |  |  |  |  |  |  | |  |
| *Zm00001d011352* | -1.8 | -1.9 | -2.5 | -2.3 |  |  |  |  |  |  |  |  | 3.8 |  |  |  |  |  |  |  |  |  |  |  |  |  |  |  |  | |  |
| **Zm00001d011721** |  |  |  | -1.1 |  |  |  |  | -1.3 | -1.1 |  |  |  |  |  |  |  |  |  |  |  |  |  |  |  |  |  |  |  | |  |
| *Zm00001d013289* |  |  |  |  |  |  | 1.1 |  |  |  |  |  |  |  |  | 2.1 | 1.5 | 1.6 | 3.2 |  |  |  |  | -1.2 |  |  |  |  |  | |  |
| Zm00001d013680 |  |  |  |  |  |  |  |  |  |  |  |  |  |  |  |  |  |  | 1.4 |  |  |  | -2.2 |  |  |  |  |  |  | |  |
| *Zm00001d016660* |  |  |  |  |  |  |  |  |  |  |  |  |  |  |  |  |  |  |  |  |  |  | 1.2 |  |  |  |  |  |  | |  |
| Zm00001d017612 |  |  |  |  |  |  |  |  |  |  | 5.0 |  |  |  |  |  |  |  |  | 1.4 |  |  |  |  |  |  |  |  |  | |  |
| *Zm00001d017908* |  |  |  |  |  |  |  |  |  |  |  |  |  |  |  |  |  |  |  |  |  |  | -2.3 |  |  |  |  | -1.7 |  | |  |
| Zm00001d018344 | -1.1 |  | -1.1 | -1.2 |  |  |  |  |  |  |  |  |  |  |  |  |  |  |  |  |  |  |  |  |  |  |  |  |  | |  |
| *Zm00001d018369* |  |  |  |  |  |  |  |  |  |  |  |  |  |  |  |  |  |  |  |  |  |  |  |  |  | -1.2 |  |  |  | |  |
| *Zm00001d019527* |  |  |  |  |  |  |  |  |  |  |  | -2.6 |  |  |  |  | -1.4 |  | -1.4 |  |  |  | -1.3 |  |  |  |  |  |  | |  |
| Zm00001d019983 |  |  |  |  |  |  |  |  |  |  | -2.3 |  | 2.2 |  |  |  |  |  |  |  |  |  |  |  |  |  |  |  |  | |  |
| Zm00001d020256 |  |  |  |  |  |  |  |  |  |  |  |  |  |  |  |  |  |  | 1.4 |  |  |  |  |  |  |  |  |  |  | |  |
| Zm00001d021839 |  |  | -1.5 | -1.2 |  |  |  |  | 3.9 |  |  |  |  | 1.1 |  |  |  |  | -2.0 |  |  | -1.3 |  |  |  |  |  |  |  | |  |
| *Zm00001d021885* |  |  |  |  |  |  |  |  | 5.7 |  |  |  |  |  |  |  |  |  |  |  |  |  |  |  |  |  |  |  |  | |  |
| Zm00001d021927 |  | -1.2 |  | -1.4 |  |  |  |  |  |  |  |  |  |  |  |  |  |  |  |  |  |  |  |  |  |  |  |  |  | |  |
| *Zm00001d022239* | -1.1 | -1.7 | -1.1 | -2.3 |  |  |  |  |  |  |  |  |  |  |  |  |  |  | -1.0 |  |  |  | -1.3 |  |  |  |  |  |  | |  |
| *Zm00001d024166* |  |  |  |  |  |  |  |  |  |  |  |  |  |  |  |  |  |  |  |  |  | 1.6 |  | 1.4 | 1.1 |  |  |  |  | |  |
| *Zm00001d024430* |  |  |  |  |  |  |  |  |  | -1.3 |  |  |  |  |  |  |  |  |  |  |  |  |  |  |  |  |  |  |  | |  |
| *Zm00001d025580* |  |  |  |  |  |  |  |  |  |  | 2.4 |  |  |  |  |  |  |  |  |  |  |  |  |  | 1.0 |  |  |  |  | |  |
| *Zm00001d025714* |  |  |  |  |  |  |  |  |  |  |  |  |  |  |  |  |  |  |  |  |  |  | -1.2 |  |  |  |  |  |  | |  |
| Zm00001d027886 |  |  | 1.0 |  |  |  |  |  |  |  |  |  |  |  |  | 1.2 |  |  |  |  |  |  |  |  |  |  |  |  |  | |  |
| Zm00001d027957 |  |  |  |  |  |  |  |  |  |  | 2.4 |  |  |  |  |  |  |  |  | 1.6 |  |  | 1.2 |  |  |  |  |  |  | |  |
| *Zm00001d028701* |  |  |  |  |  |  |  |  |  |  |  |  |  |  |  |  |  |  |  |  |  |  |  |  |  |  | -1.1 |  |  | |  |
| Zm00001d029597 |  |  |  |  |  |  |  |  |  |  |  | -2.0 |  |  |  |  |  |  |  |  |  |  |  |  |  |  |  |  |  | |  |
| *Zm00001d029785* |  |  |  |  |  |  | 1.0 |  | 1.1 |  |  |  |  | 1.6 |  |  |  |  |  |  |  |  |  |  |  |  |  |  |  | |  |
| Zm00001d031088 |  | 1.1 | 1.1 | 1.3 |  |  |  |  |  |  |  |  |  |  |  |  |  |  |  |  |  |  |  |  |  |  |  |  |  | |  |
| Zm00001d033217 |  |  |  |  |  |  |  |  |  |  |  |  |  |  |  |  |  |  | 1.4 |  |  |  | -1.9 |  |  |  |  |  |  | |  |
| Zm00001d033228 | 1.0 | 1.6 | 1.9 | 2.0 |  |  | 1.0 |  |  |  |  |  |  |  |  |  |  |  |  |  |  |  |  |  |  |  |  |  |  | |  |
| *Zm00001d033990* |  |  |  |  | 1.9 |  | 3.1 |  |  |  |  |  | -4.1 |  |  | 1.9 | 3 | 2.3 | 4.7 |  |  | 2.2 | 5.3 |  |  | 2.2 |  |  |  | |  |
| *Zm00001d034817* |  |  |  |  |  |  |  |  |  |  |  |  |  |  |  |  |  |  |  |  |  |  | -1.4 |  |  |  |  |  |  | |  |
| *Zm00001d037950* |  |  | 1.1 |  |  |  |  |  |  |  |  |  |  |  |  |  |  |  | 1.2 |  |  |  |  |  |  |  |  |  |  | |  |
| Zm00001d038195 |  |  |  |  |  |  |  |  |  |  |  |  |  | 1.1 |  |  |  |  | -1.1 |  |  |  | -2.3 |  |  |  |  |  |  | |  |
| *Zm00001d039362* | -1.4 | -1.3 | -1.7 | -1.7 |  |  |  |  |  |  |  |  |  |  |  |  |  |  |  |  |  |  |  |  |  |  |  |  |  | |  |
| Zm00001d039439 |  |  |  |  |  |  |  |  |  |  |  |  |  |  |  |  |  |  | -1.3 |  |  |  |  |  |  |  |  |  |  | |  |
| *Zm00001d040423* |  |  |  |  |  |  |  |  |  |  |  |  |  |  |  |  |  |  |  |  |  |  | 2.6 |  |  |  |  |  |  | |  |
| Zm00001d041307 |  |  |  | 1.0 |  |  |  |  |  |  |  |  |  |  |  |  |  |  |  |  |  |  |  |  |  |  |  |  |  | |  |
| *Zm00001d044142* | 2.5 | 1.8 | 2.9 | 2.0 | 1.2 |  | 2.8 |  | 4.9 |  | 2.4 |  |  | 1.8 | 1.7 | 1.5 |  |  | 1.1 | 3.1 |  |  |  |  |  |  |  |  |  | |  |
| *Zm00001d044479* |  |  |  |  |  |  |  |  |  |  | 3.9 |  | 2.1 |  |  |  |  |  |  |  |  |  |  |  |  |  |  |  |  | |  |
| Zm00001d045507 |  |  |  |  |  |  | -1.1 |  |  |  |  |  |  |  |  |  |  |  |  |  |  |  |  |  |  |  |  |  |  | |  |
| Zm00001d045568 |  |  |  |  |  |  |  |  |  | -1.5 |  |  |  |  |  |  |  |  |  |  |  |  |  |  |  |  |  |  |  | |  |
| Zm00001d046305 |  |  |  |  |  |  |  |  |  | -1.2 |  |  |  |  |  |  |  |  |  |  |  |  |  |  |  |  |  |  |  | |  |
| Zm00001d047053 |  |  |  | -1.1 |  |  |  |  |  |  |  |  |  |  |  |  |  |  |  |  |  |  |  |  |  |  |  |  |  | |  |
| Zm00001d047842 |  | -1.2 | -1.0 | -1.7 |  |  |  |  |  |  |  |  |  |  |  |  |  |  |  |  |  |  |  |  |  |  |  |  |  | |  |
| *Zm00001d048345* |  |  |  |  |  |  |  |  | 1.3 |  |  |  |  |  |  |  |  |  |  |  |  |  |  |  |  |  |  |  |  | |  |
| Zm00001d048404 |  |  |  |  |  |  |  |  |  |  |  |  | 4.3 |  |  |  |  |  | 1.2 |  |  |  | -1.5 |  |  |  |  |  |  | |  |
| *Zm00001d048868* | 1.5 |  | 1.6 |  |  |  |  |  |  |  |  |  | -3.3 |  |  | 1.1 |  | 1.0 |  |  |  |  |  |  |  |  |  |  |  | |  |
| Zm00001d050132 |  |  |  |  |  |  | 1.3 |  |  |  |  |  |  |  |  |  |  |  | 1.1 |  |  |  |  |  |  |  |  |  |  | |  |
| *Zm00001d050635* |  |  |  |  |  |  |  |  |  |  | -1.7 | -2.0 |  |  |  |  |  |  |  |  |  |  |  |  |  |  |  |  |  | |  |
| *Zm00001d050868* |  |  |  | -1.0 |  |  |  |  | 1.3 |  |  |  |  |  |  |  |  |  | -1.0 |  |  |  |  |  |  |  |  |  |  | |  |
| *Zm00001d052054* |  |  |  |  |  |  |  |  |  | -1.6 |  |  |  |  |  |  |  |  |  | 1.2 |  |  |  |  |  |  |  |  |  | |  |
| Zm00001d052323 |  |  |  |  |  |  | -1.2 |  |  |  |  |  |  |  |  |  |  |  |  | -1.1 |  |  |  |  |  |  |  |  |  | |  |
| *Zm00001d052423* |  |  |  |  |  |  |  |  |  |  |  |  |  |  |  |  |  |  |  |  |  |  | -1.5 |  |  |  |  |  |  | |  |
| Zm00001d052944 |  |  |  |  |  |  |  |  |  |  |  |  |  | 1.8 |  |  |  |  |  |  |  |  |  |  |  |  |  |  |  | |  |
| Zm00001d053725 |  |  |  |  |  |  |  |  |  | -1.1 |  |  |  |  |  |  |  |  |  |  |  |  |  |  |  |  |  |  |  | |  |

**Reference list for this file:**

Zheng J, Fu J, Gou M, Huai J, Liu Y, Jian M, et al. Genome-wide transcriptome analysis of two maize inbred lines under drought stress. Plant Mol Biol. 2010; 72: 407-421.

Opitz N, Paschold A, Marcon C, Malik WA, Lanz C, Piepho HP et al. Transcriptomic complexity in young maize primary roots in response to low water potentials. BMC Genomics. 2014; 15: 741.

Ding Y, Virlouvet L, Liu N., Riethoven JJ, Fromm M, Avramova Z. Dehydration stress memory genes of *Zea mays*; comparison with *Arabidopsis thaliana*. BMC Plant Biol. 2014; 14: 141.

Liu Y, Zhou M, Gao Z, Ren W, Yang F, He H, et al. RNA-Seq Analysis Reveals MAPKKK Family Members Related to Drought Tolerance in Maize. PloS ONE. 2015; 10: e143128.

Forestan C, Cigliano RA, Farinati S, Lunardon A, Sanseverino W, Varotto S. Stress-induced and epigenetic mediated maize transcriptome regulation study by means of transcriptome reannotation and differential expression analysis. Sci Rep. 2016; 6: 30446.

***^1^*** Thatcher SR, Danilevskaya ON, Meng X, Beatty M., Zastrow-Hayes G, Harris C., et al. Genome-wide analysis of alternative splicing during development and drought stress in maize. Plant Physiol. 2016; 170: 589-599.

Zhang X, Liu X, Zhang D, Tang H, Sun B, Li C, Hao L, et al. Genome-wide identification of gene expression in contrasting maize inbred lines under field drought conditions reveals the significance of transcription factors in drought tolerance. PLoS ONE. 2017; 12: e0179477.

*^1^ This study was also re-analyzed by: Miao Z, Han Z, Zhang T, Chen S, Ma C. A systems approach to a spatiotemporal understanding of the drought stress response in maize. Sci Rep. 2017; 7: 6590.*
