# Supplementary figures and images for: Drought-tolerant and drought-sensitive genotypes of maize (Zea mays L.) differ in contents of endogenous brassinosteroids and their drought-induced changes
Source: PLoS One. 2018 May 24;13(5):e0197870. doi: 10.1371/journal.pone.0197870 (PMC5967837; doi:10.1371/journal.pone.0197870)

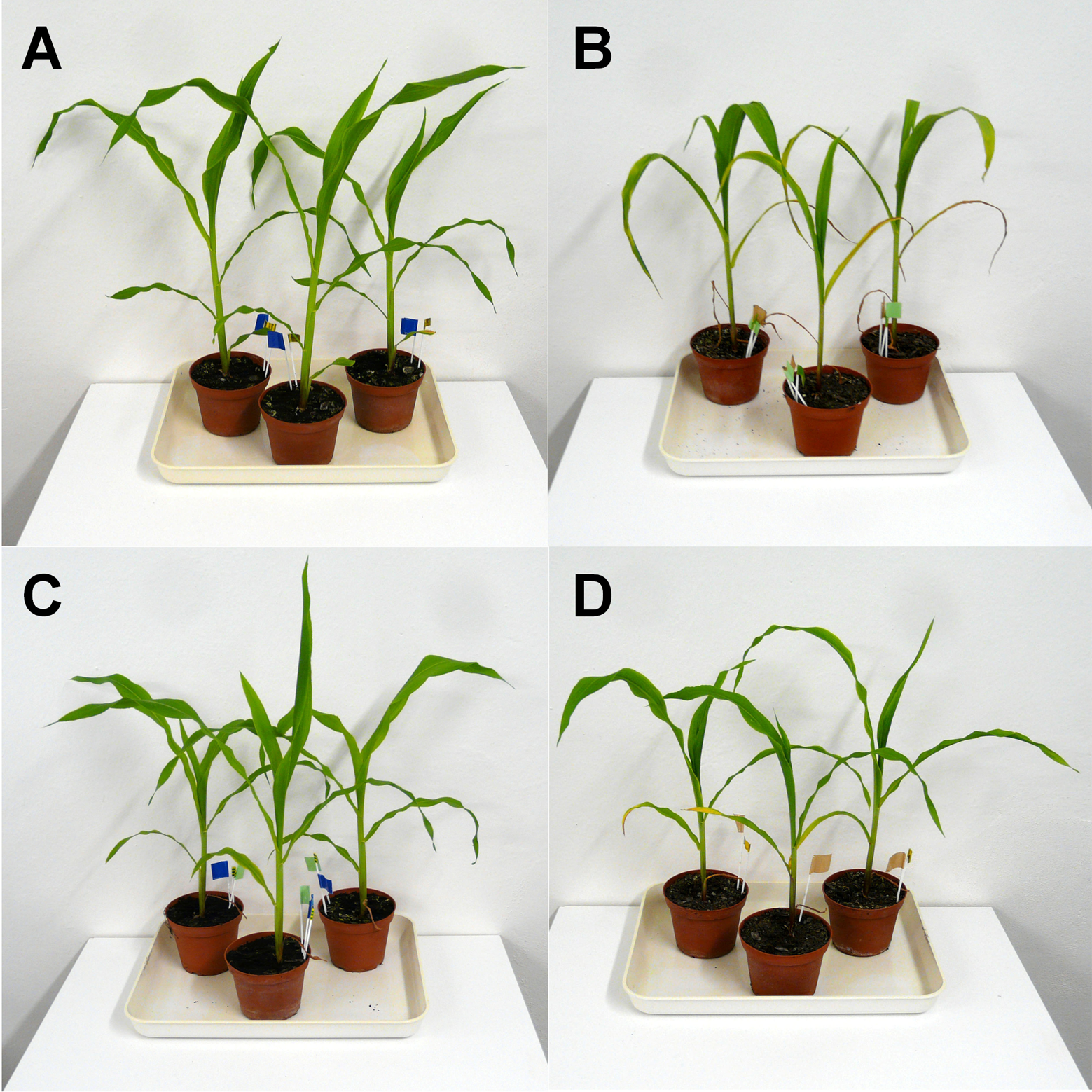

Supplement: S1 Fig — Phenotypic representation of two maize genotypes, 2023 (A, B) and CE704 (C, D). Plants were subjected either to normal watering (control; A, C) or to 14 days of withholding water (stress; B, D). (TIF) [file pone.0197870.s005.tif]
